# Angptl5 restricts primitive hematopoiesis by promoting retinoic acid signaling in zebrafish

**DOI:** 10.1371/journal.pbio.3003858

**Published:** 2026-06-25

**Authors:** Jing Mo, Ding-Hao Zhuo, Min Gao, Ying Huang, Tao Cheng, Yang Dong, Yan-Yi Xing, Yun-Fei Li, Zi-Xin Jin, Xiang Liu, Guo-Qin Zhao, Hai-Rong Pu, Yu-Meng Liu, Li-Ping Shu, Peng-Fei Xu

**Affiliations:** 1 Department of Immunology, School of Basic Medicine, Guizhou Medical University, Guiyang, Guizhou, China; 2 Center for Genetic Medicine, the Fourth Affiliated Hospital of School of Medicine, and International School of Medicine, Zhejiang University, Yiwu, China; 3 Institute of Genetics, Zhejiang University School of Medicine, Hangzhou, Zhejiang, China; 4 Women’s Hospital, Zhejiang University School of Medicine, Hangzhou, Zhejiang, China; 5 National & Guizhou Joint Engineering Laboratory for Cell Engineering and Biomedicine Technique, and Guizhou Province Key Laboratory for Regenerative Medicine, Guizhou Medical University, Guiyang, Guizhou, China; University of Pennsylvania School of Medicine, UNITED STATES OF AMERICA

## Abstract

Homeostasis is essential for hematopoiesis, and its dysregulation can lead to severe pathological conditions. Retinoic acid (RA) is a key regulator that exerts concentration-dependent effects on both embryonic and adult hematopoiesis. However, the mechanisms that modulate RA signaling in hematopoietic processes remain poorly understood. Using zebrafish as a model, we identified angiopoietin-like protein 5 (Angptl5) as a critical regulator of hematopoietic homeostasis. Loss of Angptl5 function resulted in myeloid hyperplasia in the anterior lateral plate mesoderm (ALPM) and anterior expansion of erythroid progenitors in the posterior lateral plate mesoderm (PLPM)—phenotypes consistent with attenuated RA signaling. Molecular analyses confirmed impaired RA signaling in *angptl5*^*Δ10/Δ10*^ mutants, and exogenous RA supplementation fully rescued the hematopoietic defects. Mechanistically, we found that Angptl5 transcriptionally activates retinol dehydrogenase *dhrs9* through its interaction with Integrin α6lβ5. Our findings establish Angptl5 as a novel and essential regulator of embryonic hematopoiesis and reveal a previously unrecognized mechanism controlling hematopoietic homeostasis. These insights position Angptl5 as a potential therapeutic target for hematological disorders.

## Abstract 2

Retinoic acid (RA) regulates hematopoietic homeostasis during development, however the mechanisms underlying RA signaling remain unclear. This study defines a Angptl5-Integrin α6lβ5-ERK axis that regulates RA signaling and restrains myeloid and erythroid progenitor expansion during zebrafish embryogenesis.

## Introduction

Primitive hematopoiesis in vertebrates, such as zebrafish, is spatially organized along the anterior-posterior (A-P) embryonic axis, with distinct progenitor populations emerging from the anterior lateral plate mesoderm (ALPM) and posterior lateral plate mesoderm (PLPM) [1–3]. The transcription factor *tal1* serves as a critical marker for the onset of the hematopoietic program, with expression initiating in the ALPM and PLPM at the 2-somite stage in zebrafish [4]. In the ALPM, subsets of *tal1*-expressing cells acquire a myeloid fate, marked by the expression of *spi1b* (a myeloid-specific transcription factor) [5]. In contrast, the PLPM exhibits bipotent erythro-myeloid potential, as evidenced by the co-expression of *spi1b* and *gata1a* (an erythroid-specific transcription factor) [6–8].

Retinoic acid (RA), a key morphogen involved in patterning the A-P axis and regulating tissue specification during embryonic development, has been shown to suppress *spi1b* expression in the ALPM [9]. Interestingly, RA inhibition causes an anterior shift of *gata1a*-positive cells [10], suggesting that RA signaling may contribute to the establishment and maintenance of the boundary between anterior and posterior hematopoietic domains [11]. In addition, RA has been successfully used to treat specific types of leukemia [12]. However, the mechanism modulating RA in orchestrating primitive hematopoiesis remains unclear.

Angiopoietin-like proteins (ANGPTLs) are a family of secreted glycoproteins involved in a variety of developmental and disease processes, including angiogenesis, HSCs maintenance, and cancer progression [13,14]. For example, ANGPTL1 suppresses the integrin signaling to inhibit angiogenesis and metastasis in hepatocellular carcinoma [15], while ANGPTL4 is essential for common myeloid progenitor differentiation and inflammatory responses [16,17]. ANGPTL5 has been shown to enhance *ex vivo* expansion of human cord blood hematopoietic stem cells (HSCs) [18] and interact with inhibitory receptors to support leukemia progression [19]; however, its function during embryonic development remains unexplored. Notably, a murine ortholog of ANGPTL5 has not been identified to date, making zebrafish a particularly relevant model for studying its developmental functions.

In this study, we used zebrafish as a model to investigate the function of Angptl5 in primitive hematopoiesis. We found that *angptl5* knockout results in myeloid hyperplasia in the ALPM and anterior expansion of erythroid progenitors in the PLPM-phenotypes reminiscent of RA deficiency. Molecular analysis revealed downregulation of RA signaling in angptl5 mutants, and RA treatment was able to rescue the hematopoietic defects. Mechanistically, we found that Angptl5 transcriptionally activates the retinol dehydrogenase *dhrs9*, a key enzyme in the RA synthesis pathway, through its interaction with integrin α6lβ5 and subsequent activation of the ERK signaling pathway. Collectively, our findings identify Angptl5 as a new regulator of primitive hematopoiesis that function upstream of RA signaling.

## Results

### *angptl5* is expressed in the primitive hematopoietic tissue during zebrafish embryonic development

**Fig 1:**
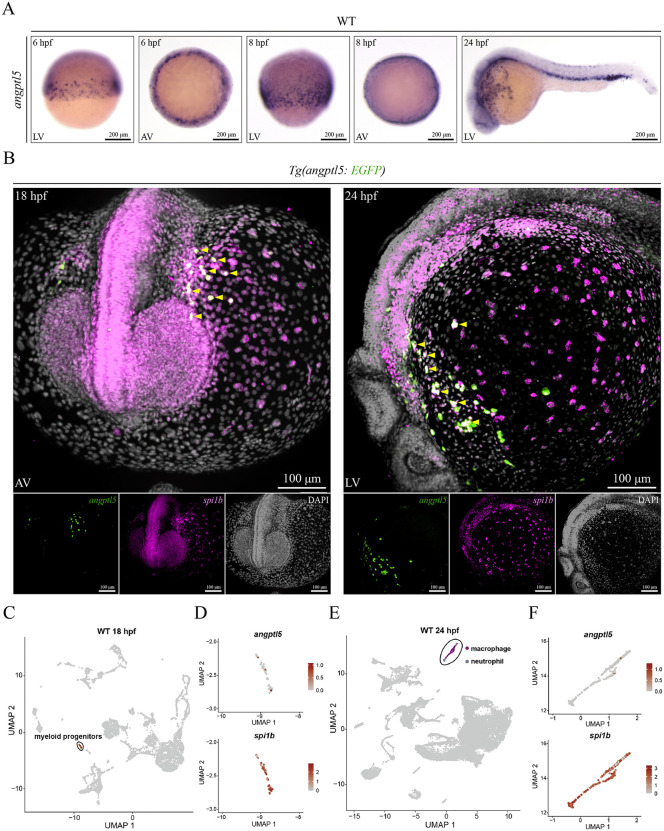
Expression pattern of *angptl5* during zebrafish embryonic development. **(A)** Whole-mount in situ hybridization (WISH) of *angptl5* in zebrafish embryos at the indicated stages. **(B)** Projection of images from confocal stacks to show co-localization of myeloid progenitor marker *spi1b* mRNA and EGFP protein in *Tg(angptl5:EGFP)*. Each imaging was performed for at least three independent replicates. **(C–F)** Co-expression of *angptl5* and *spi1b* in single-cell analysis. UMAP analysis of 18 hpf (C) and 24 hpf (E) WT zebrafish embryos from the published dataset [20], with myeloid lineage cells highlighted. Feature plots showing the expression of *angptl5* and *spi1b* in myeloid progenitors (D) and in myeloid lineages (F). Abbreviations: hpf, hours post-fertilization; LV, lateral view; AV, animal view.

Angiopoietin-like proteins are secreted glycoproteins conserved across vertebrates (S1A and S1B Fig). Members of this family feature two highly conserved domains: an N-terminal coiled-coil domain (CCD) and a C-terminal fibrinogen-like domain (FLD) (S1C Fig). To investigate their potential roles in development, we first analyzed the spatial and temporal expression patterns of angiopoietin-like genes during zebrafish embryogenesis using whole-mount in situ hybridization (WISH). Interestingly, different angiopoietin-like genes exhibited distinct tissue specificity. For example, *angptl2b* was expressed in the posterior spinal cord, *angptl6* in the notochord, and *angptl7* in the somites (S2A Fig). Among these, *angptl5* was expressed in the mesendoderm during gastrulation and specifically enriched in hematopoietic tissues at 24 hours post-fertilization (hpf), including the rostral blood island (RBI) and posterior intermediate cell mass (ICM) (Figs 1A and S2B and S2C). To further confirm its expression, we generated a transgenic line *Tg*(*angptl5:EGFP*) by cloning the upstream 867 bp region of *angptl5* gene and EGFP coding sequece, which revealed expression of *angptl5* in the ALPM and partial co-localization with *spi1b*-positive cells (Figs 1B and S2D). Additionally, analysis of a single-cell RNA sequencing (scRNA-seq) dataset showed that *angptl5* is expressed in a subset of *spi1b*-positive cells at both 18 hpf and 24 hpf (Fig 1C–1F) [20]. Together, these findings suggest that *angptl5* may play a role in primitive hematopoiesis in zebrafish.

### *angptl5* mutation leads to hyperplasia of myeloid and erythroid progenitors

To investigate the function of Angptl5 in hematopoiesis, we generated two *angptl5* mutant lines using CRISPR/Cas9: *angptl5*Δ*10* (10 bp deletion) and *angptl5*Δ*5* (5 bp deletion) (S3A Fig). Both mutations induced frameshifts producing premature stop codons, resulting in truncated proteins that either lacked the FLD(Angptl5Δ10) or both the CCD and FLD (Angptl5Δ5).

**Fig 2:**
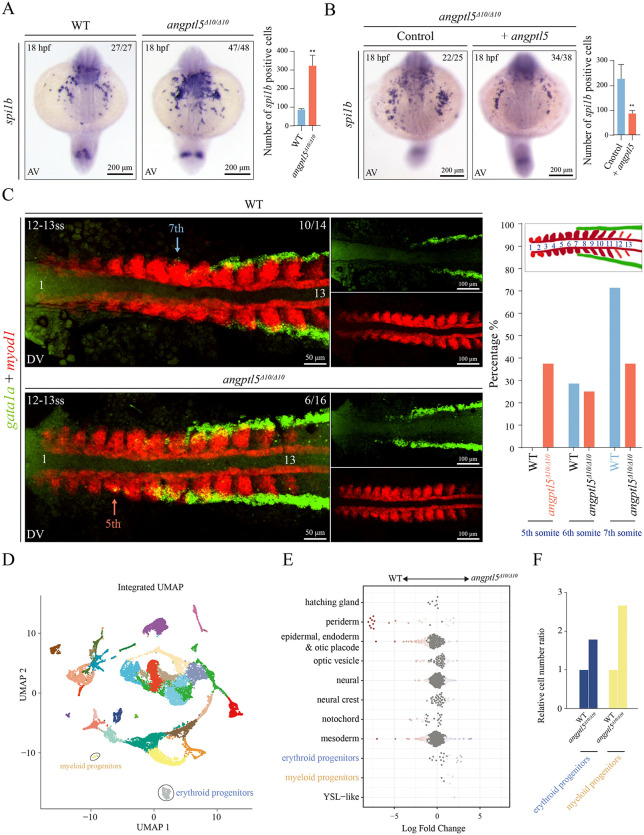
Angptl5 deficiency impairs primitive hematopoiesis. **(A)** WISH of *spi1b* in WT and *angptl5*^*Δ10/Δ10*^ embryos. Data presented as the mean ± SD, n(WT) = 27, n(*angptl5*^*Δ10/Δ10*^) = 48. Statistical significance: **P < 0.01 (Unpaired *t t*est). **(B)** WISH of *spi1b* in *angptl5*^*Δ10/Δ10*^ embryos injected with *angptl5* mRNA at the 1-cell stage. Uninjected embryos were used as a control. Data presented as the mean ± SD, n(WT) = 25, n(*angptl5*^*Δ10/Δ10*^) = 38. Statistical significance: **P < 0.01 (Unpaired *t* tes*t*). **(C)** Projection of images from confocal stacks to show *gata1a* and *myod1* expression in flat-mounted embryos in WT and *angptl5*^*Δ10/Δ10*^ embryos as assessed using HCR. Statistics are shown on the right. n(WT) = 14, n(*angptl5*^*Δ10/Δ10*^) = 16. **(D)** Integrative UMAP analysis of 16 hpf WT and *angptl5*^*Δ10/Δ10*^ embryos. The WT dataset is from the published work [21]. Each cell is coloured according to cell type annotations. **(E)** Bees warm plot showing the differential abundance by cell types between WT and *angptl5*^*Δ10/Δ10*^ embryos. **(F)** Bar plot showing the relative cell number ratios of each hematopoietic subtype to the total cell count in *angptl5*^*Δ10/Δ10*^ embryos compared to WT embryos. LV, lateral view; AV, anterior view. The data for this figure can be found in S1 Data.

While expression of hemangioblasts marker *tal1* appeared normal, *angptl5*Δ*10* mutants displayed significant expansion of *spi1b*+ myeloid progenitors in the ALPM (Fig 2A), along with increased neutrophils (*lyz*+, *mpx*+) and macrophages (*mpeg1.1*+) (S3C Fig). This phenotype was consistently observed in *angptl5*Δ*5* mutants (S3D and S3E Fig), suggests that loss of angptl5 function induces defects in primitive myelopoiesis. Based on these findings, the *angptl5*Δ*10* mutant was selected for further functional analysis. Markedly, ectopic *angptl5* mRNA expression effectively suppressed the aberrant expansion of *spi1b+* myeloid progenitor in *angptl5*^*Δ10/Δ10*^ embryos (Fig 2B). We then checked the expression pattern of *gata1a*+ erythroid progenitors. Interestingly, HCR co-staining with the somite marker *myod1* revealed anterior expansion of *gata1a* (Fig 2C), a phenotype resembling attenuated RA signaling [10]. And quantitative real-time PCR (qPCR) also confirmed upregulation of *spi1b* and *gata1a* in *angptl5*^*Δ10/Δ10*^ embryos, while *tal1* and the angioblast marker *etsrp* remained unchanged (S3F Fig). Collectively, these data define a specific primitive hematopoietic defect in *angptl5* mutants. Importantly, this defect was not associated with significant embryonic lethality under standard conditions, as determined by systematic survival analysis (S3G Fig).

To further characterize the hematopoietic phenotype caused by Angptl5 deficiency, we performed scRNA-seq on 16 hpf *angptl5*^*Δ10/Δ10*^ mutant embryos and integrated these data with published WT scRNA-seq datasets [21]. While UMAP visualization revealed similar overall cell type distributions between mutant and WT embryos (Figs 2D, S4A and S4B), differential abundance analysis identified significant expansion of hematopoietic populations in mutants, including erythroid progenitors and myeloid progenitors (Fig 2E and 2F).

Angiopoietin-like proteins have been previously reported to play roles in angiogenesis [22,23]. To assess whether *angptl5* affects vascular development, we first examined endothelial precursors during the critical stages of primitive hematopoiesis. The expression of key markers, including the vascular progenitor marker *etsrp* [24] and *fli1* [25], in the ALPM and PLPM was comparable between mutant and WT embryos (S5A and S5B Fig). Then, to evaluate potential defects at later stage, we analyzed the endothelial marker *kdrl* [26] as well as *etsrp* again at 3 dpf and 3.5 dpf using WISH and transgenic reporter lines. No obvious vascular alterations were observed in *angptl5*^*Δ10/Δ10*^ embryos at these later stages (S5C and S5D Fig).

Taken together, the main phenotypes observed in *angptl5* mutants are myeloid hyperplasia in the ALPM and anterior expansion of erythroid progenitors in the PLPM, while the vasculogenesis remains largely unchanged.

### *angptl5* mutation leads to attenuated RA signaling

**Fig 3:**
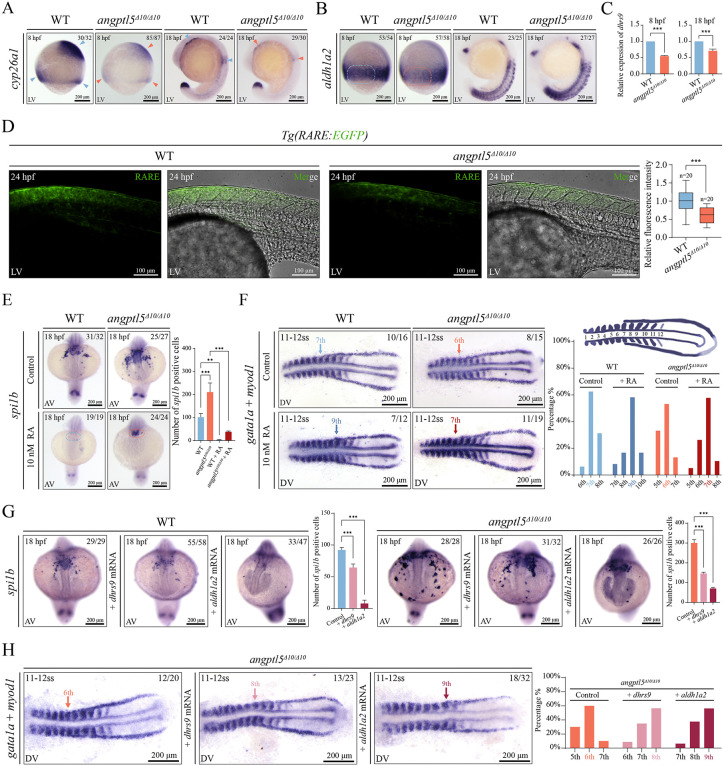
*angptl5* loss-of-function attenuates retinoic acid (RA) signaling. (**A** and **B**) WISH of *cyp26a1* (A) and retinal dehydrogenase *aldh1a2* (B) in WT and *angptl5*^*Δ10/Δ10*^ embryos. (**C**) Quantitative real-time PCR (qPCR) shows the retinol dehydrogenase *dhrs9* mRNA expression in WT and *angptl5*^*Δ10/Δ10*^ embryos. Data presented as the mean ± SD, *n* = 30. Three independent biological replicates were used. Statistical significance: ***P < 0.001 (Unpaired *t t*est). (**D**) RA signaling in WT and *angptl5*^*Δ10/Δ10*^ embryos shown by *RARE:EGFP* reporter. Statistics are shown on the right. Data presented as the mean ± SD, *n* = 20. Statistical significance: ***P < 0.001 (Unpaired *t* tes*t*). (**E**) WISH of *spi1b* in WT and *angptl5*^*Δ10/Δ10*^ embryos treated with RA. Untreated embryos were used as control. Statistics are shown on the right. Data presented as the mean ± SD, n(WT) = 32, n(*angptl5*^*Δ10/Δ10*^) = 27, n(WT + RA) = 19, n(*angptl5*^*Δ10/Δ10*^ + RA) = 24. Statistical significance: **P < 0.01, ***P < 0.001 (One-way ANOVA). (**F**) WISH of *gata1a* and *myod1* in flat-mounted WT and *angptl5*^*Δ10/Δ10*^ embryos treated with RA from the shield stage to the 11–12 somite stage (ss). Statistics are shown on the right. n(WT) = 16, n(*angptl5*^*Δ10/Δ10*^) = 15, n(WT + RA) = 12, n(*angptl5*^*Δ10/Δ10*^ + RA) = 19. (**G**) WISH of *spi1b* in WT and *angptl5*^*Δ10/Δ10*^ embryos injected with *dhrs9* or *aldh1a2* mRNA at the 1-cell stage. Uninjected embryos were used as control. Statistics are shown on the right of the representative photos. Statistics are shown on the right. Data presented as the mean ± SD, n(WT) = 29, n(WT+*dhrs9*) = 58, n(WT+*aldh1a2*) = 47, n(*angptl5*^*Δ10/Δ10*^) = 28, n(*angptl5*^*Δ10/Δ10*^ + *dhrs9*) = 32, n(*angptl5*^*Δ10/Δ10*^ + *aldh1a2*) = 26. Statistical significance: ***P < 0.001 (One-way ANOVA). (**H**) WISH of *gata1a* and *myod1* in flat-mounted WT and *angptl5*^*Δ10/Δ10*^ embryos injected with *dhrs9* or *aldh1a2* mRNA at the 1-cell stage. Statistics are shown on the right. n(*angptl5*^*Δ10/Δ10*^) = 20, n(*angptl5*^*Δ10/Δ10*^ + *dhrs9*) = 23, n(*angptl5*^*Δ10/Δ10*^ + *aldh1a2*) = 32. LV, lateral view; AV, anterior view; DV, dorsal view. The data for this figure can be found in S1 Data.

In addition to the hematopoietic defects in *angptl5* mutants, characterized by elevated *spi1b* and anterior expansion of *gata1a* that resemble the phenotype observed upon RA signaling inhibition in zebrafish [10] and mirror vitamin A deficiency-induced promotion of hematopoietic stem cell differentiation in mice [27], we also observed mesoderm involution defects during gastrulation, as indicated by aberrant *fn1a* [28] expression detected via WISH (S6A Fig), reflecting mesoderm involution defects reminiscent of those observed under RA-deficient conditions [29]. Therefore, to determine whether RA signaling is affected in *angptl5* mutants, we first examined the expression of RA direct target *cyp26a1* [30], as well as two key enzymes required for RA biosynthesis: retinol dehydrogenase *dhrs9* and aldehyde dehydrogenase *aldh1a2* (S6B Fig). Interestingly, WISH and qPCR analyses revealed significantly reduced expression of *cyp26a1* at 8 hpf and 18 hpf, *aldh1a2* at 8 hpf, and *dhrs9* at 8 hpf and 18 hpf in *angptl5*^*Δ10/Δ10*^ embryos (Fig 3A–3C). To further validate these findings, we utilized the *Tg(RARE:EGFP*) transgenic zebrafish line, in which EGFP expression is driven by RA response elements, and thus serves as a direct readout of RA signaling activation [31]. We observed *angptl5*^*Δ10/Δ10*^ embryos exhibited remarkably reduced EGFP fluorescence compared to WT embryos, providing further evidence of attenuated RA signaling in *angptl5* mutants (Figs 3D and S6C).

To investigate the hierarchy relation between Angptl5 and RA signaling in hematopoiesis regulation, we treated WT and *angptl5*^*Δ10/Δ10*^ embryos with RA inhibitor (DEAB, inhibitor of *aldh1a2*) or RA at varying concentrations starting from shield stage. As shown by the results, RA exhibited dose-dependent teratogenic effects, with concentrations ≥100 nM causing severe morphological abnormalities in WT embryos (S6D Fig) at 18 hpf. Pharmacological inhibition of RA markedly suppressed *cyp26a1* expression, whereas exogenous RA administration robustly upregulated this RA target gene in a dose-dependent manner (S6E Fig). While RA treatment suppressed *spi1b* expression in all embryos, *angptl5* mutants demonstrated significantly higher resistance to RA treatment, as evidenced by persistent *spi1b* and *gata1a* expression compared to WT controls (Figs 3E, 3F and S7A). Consistently, treatment with the RA receptor antagonist AGN 193109 (AGN) or DEAB led to a significant expansion of *spi1b*^+^ and *gata1a*^+^ cells in both WT and *angptl5*^*Δ10/Δ10*^ embryos (S7B and S7C Fig).

The above results suggest that RA activation could compensate for Angptl5 deficiency. To further confirm the effect of restoring RA signaling on primitive hematopoiesis in *angptl5* mutants, we performed rescue experiments by injecting *dhrs9* or *aldh1a2* mRNA at the 1-cell stage in both WT and *angptl5*^*Δ10/Δ10*^ embryos. Overexpression of both *dhrs9* and *aldh1a2* enhances the *cyp26a1* expression, indicating the elevated RA signaling (S7D Fig). Consistently, the expanded expression of *spi1b*^+^ myeloid and *gata1a*^*+*^ erythroid progenitors in *angptl5* mutants was rescued (Fig 3G and 3H). Together, these results indicate that Angptl5 function as an upstream regulator of RA-mediated hematopoietic patterning.

### Angptl5 physically interacts with integrin α6lβ5

**Fig 4:**
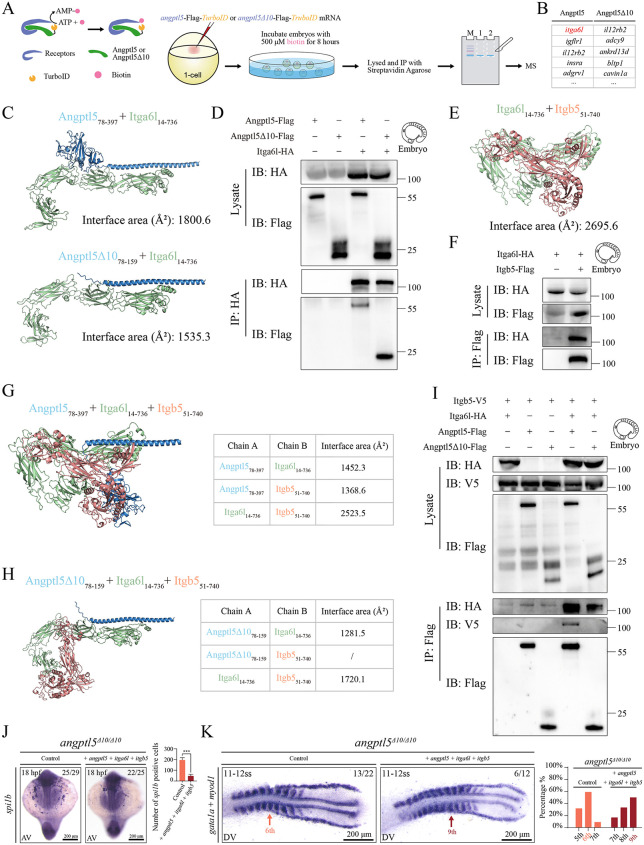
Angptl5 physically interacts with Integrin α6l and β5. **(A** and **B)** Candidate receptors of Angptl5 as assessed using immunoprecipitation-mass spectrometry (IP-MS). Schematic workflow of experimental setup for IP-MS (A) and identified proteins in Angptl5 and Angptl5Δ10 embryos were listed (B). **(C)** Structural models of the Angptl5_78-397_-Itga6l_14-736_ and Angptl5Δ10_78-159_-Itga6l_14-736_ complexes. **(D)** Interaction of Angptl5-Flag/Angptl5Δ10-Flag with Itga6l-HA in zebrafish embryos as assessed using co-immunoprecipitation (Co-IP). **(E)** Structural models of the Itga6l_14-736_-Itgb5_51-740_ complex. **(F)** Interaction of Itgb5-Flag with Itga6l-HA in zebrafish embryos as assessed using Co-IP. **(G** and **H)** Structural models of the Angptl5_78-397_-Itga6l_14-736_-Itgb5_51-740_ (G) and Angptl5Δ10_78-159_-Itga6l_14-736_-Itgb5_51-740_ (H) complexes. **(I)** Interaction of Angptl5-Flag/Angptl5Δ10-Flag, Itgb5-V5, and Itga6l-HA in zebrafish embryos as assessed using Co-IP. **(J)** WISH of *spi1b* in *angptl5*^*Δ10/Δ10*^ embryos injected with *angptl5 + itga6l + itgb5* mRNA at the 1-cell stage. Uninjected embryos were used as control. Statistics are shown on the right. Data presented as the mean ± SD, n(*angptl5*^*Δ10/Δ10*^) = 29, n(*angptl5*^*Δ10/Δ10*^ + *angptl5* + *itga6l*+*itgb5*) = 25. Statistical significance: ***P < 0.001 (Unpaired *t t*est). **(K)** WISH of *gata1a* and *myod1* in flat-mounted *angptl5*^*Δ10/Δ10*^ embryos injected with *angptl5 + itga6l + itgb5* mRNA at the 1-cell stage. Statistics are shown on the right. n(*angptl5*^*Δ10/Δ10*^) = 22, n(*angptl5*^*Δ10/Δ10*^ + *angptl5* + *itga6l*+*itgb5*) = 12. The three-dimensional structures of all protein complexes were predicted using AlphaFold3 via the AlphaFold Server (https://alphafoldserver.com). The top-ranked model based on the predicted template modeling score was selected for further analysis. All structural figures were generated using the open-source version of PyMOL. AV, anterior view; DV, dorsal view. The data for this figure can be found in S1 Data.

As a secreted protein, Angptl5 requires receptor binding to exert its biological functions. To comprehensively identify receptors through which Angptl5 regulates RA signaling, we employed TurboID-mediated proximity labeling combined with immunoprecipitation-mass spectrometry (IP-MS) [32] in zebrafish. We engineered a construct expressing *angptl5* mRNA fused with a C-terminal Flag-tagged TurboID. Secretion of engineered proteins was detected in HEK293T conditioned medium and the expression of tagged *angptl5* and biotinylated proteins was detected in zebrafish embryos (S8A and S8B Fig). The engineered *angptl5* mRNA exhibited similar rescue efficiencies to untagged controls, indicating that the fusion tags do not compromise Angptl5 protein functionality (S8C Fig). We then injected engineered WT and mutated *angptl5* mRNA into 1-cell stage embryos, and incubated those embryos with biotin for 8 hours before collecting them for IP-MS, respectively (Fig 4A).

Comparative proteomic profiling of WT versus mutant samples, we identified integrin α6-like (Itga6l) as a potential candidate, based on its specific interaction with WT Angptl5 but not the Angptl5Δ10 mutant (Fig 4B and S1 Dataset). This aligns with established literatures demonstrating that ANGPTL family members engage various integrins-conserved transmembrane proteins that regulate biochemical signaling and mechanotransduction via force-dependent conformational changes [13,33]. Structural modeling of the Angtpl5-Itga6l complex using AlphaFold3 also revealed a considerable interface area for the WT Angptl5 (Fig 4C, up) and co-immunoprecipitation (Co-IP) assay confirmed that Angptl5 can physically associate with Itga6l (Fig 4D). Notably, overexpression of *angptl5* mRNA in *angptl5*^*Δ10/Δ10*^ embryos effectively suppressed the pathological expansion of *spi1b*^+^ myeloid progenitors, but this rescue phenotype was abrogated by co-injection with itga6l morpholino (MO), indicating that Angptl5-mediated regulation of primitive hematopoiesis is dependent on Itga6l function (S8D Fig). Conversely, the Angptl5Δ10 exhibited a reduction interface area, indicating a reduced binding affinity for the truncated Angptl5 (Fig 4C, down). However, to our surprise, the Co-IP assay showed that both Angptl5 and Angptl5Δ10 can physically associate with Itga6l (Fig 4D). These findings suggest that the loss-of-function phenotype cannot be attributed solely to disrupted binding of Angptl5 to Itga6l.

Functional integrin signaling requires the formation of α/β heterodimeric complexes at the cell membrane [34]. We thus speculate that the mutation of Angptl5 may have disrupted the formation of this potential complex. To identify the β subunit partner for Itga6l in this context, we systematically evaluated potential candidates. Bioinformatic screening of existing transcriptome datasets revealed differential expression profiles across 12 integrin β subunit genes in zebrafish [35]. Among these, five subunits (*itgb1a*, *itgb1b*, *itgb4*, *itgb5*, and *itgb6*) showed detectable expression during primitive hematopoiesis (S9A Fig). Thus, we performed structural modeling of the complexes formed between Itga6l and each individual β subunit. The Itga6l-Itgb4 and Itga6l-Itgb5 complexes exhibited the two largest interfacial contact areas among all Itga6l-β subunit complexes (Figs 4E and S9B–S9F). However, the Angptl5-Itga6l-Itgb4 ternary complex failed to maintain structural stability due to incomplete pairwise interaction interfaces (S9G Fig). Instead, structural modeling demonstrated that Itgb5 engages in an extensive interaction with Itga6l, burying a surface area of 2695.6 Å² at their interface (Fig 4E), and this is further confirmed biochemically by Co-IP (Fig 4F). WISH analysis showed that both *itga6l* and *itgb5* are expressed during early development (S9H and S9I Fig).

We therefore characterized the putative Angptl5-Itga6l-Itgb5 ternary complex. Structural modeling revealed that WT Angptl5 formed stable ternary interactions through extensive interfacial contact surfaces, whereas the Angptl5Δ10 mutant exhibited disrupted binding interfaces with insufficient pairwise interaction networks (Fig 4G and 4H). Intriguingly, Co-IP assays demonstrated that Angptl5 cannot bind Itgb5 directly in the absence of Itga6l (Fig 4I). However, when Itga6l is present, WT Angptl5, but not the Δ10 mutant, mediates formation of a stable complex with Itgb5 (Fig 4I). These findings demonstrate that Angptl5 facilitates assembly of Itga6l-Itgb5 heterodimers, and that this molecular activity is abolished in Angptl5Δ10 mutant.

To elucidate the functional role of the Angptl5/Integrin α6lβ5 complex in zebrafish primitive hematopoiesis, we performed loss-of-function and gain-of-function analyses. In WT embryos, individual knockdown of *itga6l* or *itgb5*, or pharmacological inhibition of integrin function, was sufficient to increase the expression of the primitive myeloid marker *spi1b* and the erythroid marker *gata1a*, phenocopying the *angptl5* mutant (S10A Fig). Conversely, either *angptl5* mRNA injection or co-injection of *itga6l* and *itgb5* mRNAs effectively suppressed *spi1b* expression, while *angptl5*Δ*10* mRNA had no significant effect in WT embryos (S10B Fig). Notably, simultaneous overexpression of all three components (*angptl5*, *itga6l*, and *itgb5*) led to a further reduction in both *spi1b* and *gata1* expression (S10C and S10D Fig). Most importantly, in *angptl5*^*Δ10/Δ10*^ mutants, this triple overexpression (*angptl5* + *itga6l* + *itgb5*) significantly reduced both the myeloid and erythroid hyperplasia (Fig 4J and 4K).

Collectively, these results suggest that integrin α6lβ5 acts as the cognate receptor for Angptl5 in hematopoietic regulation, and that the Angptl5-mediated suppression of primitive hematopoietic progenitor cells is mechanistically dependent on integrin α6lβ5 signaling.

### ERK signaling is required for Angptl5-mediated regulation of primitive hematopoiesis

**Fig 5:**
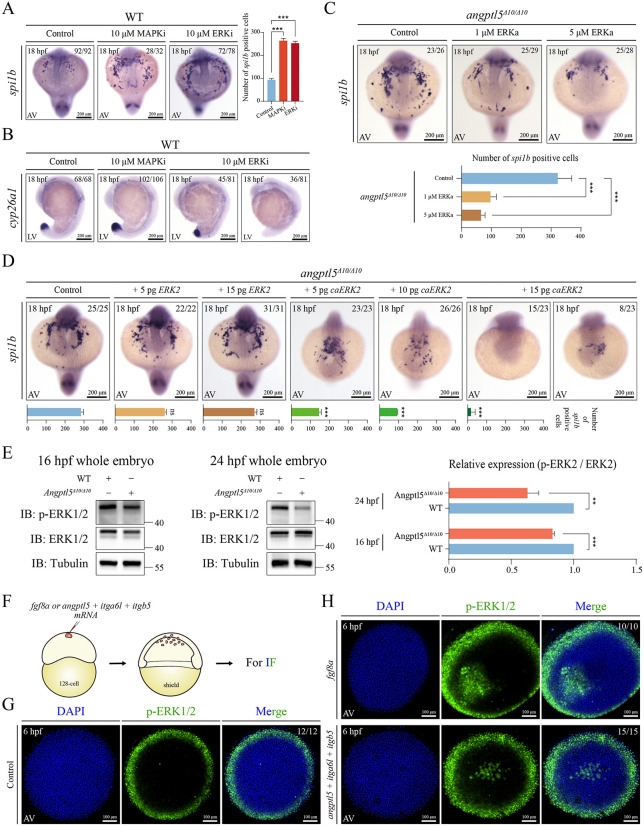
Angptl5-integrin α6lβ5 functions through the ERK signaling pathway. **(A** and **B)** WISH of *spi1b* (A) and *cyp26a1* (B) in WT embryos treated with MAPK inhibitor or ERK inhibitor from the shield stage to the 18-somite stage. Untreated embryos were used as control. Statistics are shown on the right. Data presented as the mean ± SD, n(WT) = 92, n(WT +MAPKi) = 32, n(WT + ERKi) = 78. Statistical significance: ***P < 0.001 (One-way ANOVA). **(C)** WISH of *spi1b* in *angptl5*^*Δ10/Δ10*^ embryos treated with ERK activator from the shield stage to the 18-somite stage. Untreated embryos were used as control. Statistics are shown below. Data presented as the mean ± SD, n(*angptl5*^*Δ10/Δ10*^) = 26, n(*angptl5*^*Δ10/Δ10*^ + 1 µM ERKa) = 29, n(*angptl5*^*Δ10/Δ10*^ + 5 µM ERKa) = 28. Statistical significance: ***P < 0.001 (One-way ANOVA). **(D)** WISH of *spi1b* in *angptl5*^*Δ10/Δ10*^ embryos injected with *ERK2* or *caERK2* mRNA at the 1-cell stage. Uninjected embryos were used as control. Statistics are shown below. Data presented as the mean ± SD, n(*angptl5*^*Δ10/Δ10*^) = 25, n(*angptl5*^*Δ10/Δ10*^ + 5 pg *ERK2*) = 22, n(*angptl5*^*Δ10/Δ10*^ + 15 pg *ERK2*) = 31, n(*angptl5*^*Δ10/Δ10*^ + 5 pg *caERK2*) = 23, n(*angptl5*^*Δ10/Δ10*^ + 10 *caERK2*) = 26, n(*angptl5*^*Δ10/Δ10*^ + 15 *caERK2*) = 23. Statistical significance: ***P < 0.001 (One-way ANOVA). **(E)** Expression of phosphorylated ERK1/2 (p-ERK1/2) in WT and *angptl5*^*Δ10/Δ10*^ embryos as assessed using western blot. Statistics are shown on the right. Three independent biological replicates were used. Statistical significance: **P < 0.01, ***P < 0.001 (Unpaired *t* test). **(F–H)** Schematic diagram of experimental se*t*up (F) for immunofluorescence (IF) of p-ERK1/2 in WT and *angptl5*^*Δ10/Δ10*^ embryos. *Fgf8a* mRNA or *angptl5 + itga6l + itgb5* mRNA (H) were injected into one blastomere on the animal pole at the 128-cell stage and then imaged at 6 hpf. Uninjected embryos (G) were used as control. AV, anterior view (A, C, D), animal view (G and H); LV, lateral view. The data for this figure can be found in S1 Data.

Integrin engagement by extracellular ANGPTL proteins typically activates intracellular signaling cascades, including FAK, NF-κB, and MAPK/ERK pathways, which regulate diverse cellular processes such as motility, proliferation, and transcriptional regulation [36–38]. To delineate the downstream effectors of Angptl5/α6lβ5 signaling in hematopoietic development, we systematically inhibited key pathways using small-molecule inhibitors from the shield stage onward, including: Defactinib (FAK), BAY 11–7082 (NF-κB), Adezmapimod (p38 MAPK), and Mirdametinib (ERK). Notably, FAK inhibition showed no significant effect on *spi1b* expression in the RBI, suggesting minimal involvement in primitive myeloid regulation (S11A Fig). And NF-κB blockade downregulated *spi1b* expression, revealing its role in promoting myeloid expansion (S11B Fig). Interestingly, MAPK or ERK inhibition significantly upregulated *spi1b* expression, and reduced *cyp26a1* expression, phenocopying *angptl5* loss-of-function mutants (Figs 5A, 5B, and S11C). Most notably, pharmacological activation of ERK signaling (C16-PAF) effectively rescued the *spi1b+* myeloid hyperplasia in *angptl5*^*Δ10/Δ10*^ mutants (Fig 5C).

The above results suggest that *angptl5* may regulate primitive hematopoiesis through the ERK signaling pathway. To further validate this hypothesis, we generated WT ERK2 and a constitutively activated ERK2 mutant (caERK2, L84P/S162D/D330N) [39] (S11D Fig), and injected mRNA of these two versions to rescue the phenotype resulting from *angptl5* loss-of-function, respectively. While WT *ERK2* had no obvious effect on *spi1b* expression, 10 pg of *caERK2* rescued *spi1b* expression in *angptl5*^*Δ10/Δ10*^ embryos to levels comparable to the WT, and higher doses of *caERK2* abolished *spi1b* expression in most *angptl5*^*Δ10/Δ10*^ embryos and in all WT embryos (Figs 5D and S11E). Mechanistically, we found that ERK signaling activity was significantly decreased in *angptl5*^*Δ10/Δ10*^ embryos compared to WT, as indicated by western blot and immunofluorescence (IF) of phosphorylated ERK1/2 (pERK1/2) (Figs 5E and S11H). Further spatial analysis of pERK distribution in hematopoietic regions revealed partially co-localization of pERK signal with *spi1b*+ myeloid progenitors in the ALPM (S11F Fig). In addition, pERK staining was detected within the ICM, a posterior blood-forming tissue derived from the PLPM, in WT embryos (S11G Fig). These data indicated that active ERK signaling was observed in both major primitive hematopoietic progenitor domains.

Next, we investigated whether the Angptl5/Integrin α6lβ5 complex is sufficient to active ERK signaling. We found that overexpression of *angptl5* alone, co-overexpression of *itga6l* and *itgb5*, or combined overexpression of *angptl5* with *itga6l* and *itgb5* enhanced ERK signaling (S11I Fig). To further validate this, we co-injected *angptl5*, *itga6l*, and *itgb5* mRNA into one blastomere of the animal pole at the 128-cell stage, and used *fgf8a*, a known canonical ERK activator [40] as a positive control (Fig 5F). Strikingly, combinatorial overexpression of *angptl5*, *itga6l*, and *itgb5* triggered ectopic ERK activation, similar to the activation pattern observed with overexpression of *fgf8a* (Fig 5G and 5H).

In summary, these findings delineate a pathway in which Angptl5 engages integrin α6lβ5 to activate ERK signaling, a process required for the regulation of primitive hematopoiesis by Angptl5.

### Angptl5/Integrin α6lβ5 potentiates RA activity via ERK-dependent *dhrs9* transcription

**Fig 6:**
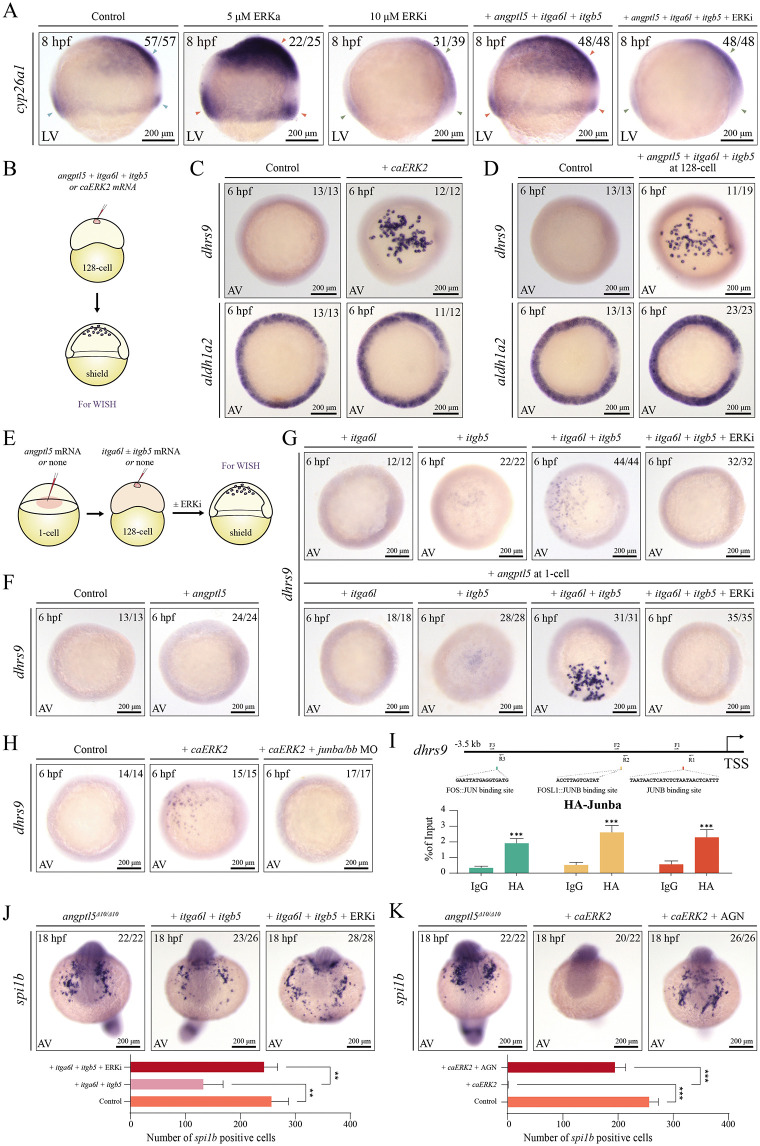
The Angptl5-integrin α6lβ5-ERK-Dhrs9-RA signaling cascade regulates primitive hematopoiesis. **(A)** WISH of *cyp26a1* in WT embryos. Embryos were treated with ERK activator (C16-PAF, ERKa) or ERK inhibitor (Mirdametinib, ERKi) from the shield stage, or injected with *angptl5* + *itga6l* + *itgb5* mRNA at the 1-cell stage. Untreated embryos were used as control. **(B–D)** Schematic diagram of experimental setup (B) for WISH of *dhrs9* and *aldh1a2* in WT embryos. *caERK2* (C) or *angptl5* + *itga6l* + *itgb5* (D) mRNA injected into one blastomere on the animal pole at the 128-cell stage and then detected at 6 hpf. **(E–G)** Schematic diagram of experimental setup (E) for WISH of *dhrs9*. WT embryos were first injected with *angptl5* mRNA at the 1-cell stage. Subsequently, *itga6l ± itgb5* mRNA was injected into one blastomere on the animal pole at the 128-cell stage. Embryos were then continuously treated with or without ERK inhibitor until the shield stage **(G)**. Uninjected embryos and only *angptl5* mRNA injected embryos (F) were used as control. **(H)** WISH of *dhrs9* in WT embryos. *caERK2* mRNA *± junba/bb* MO injected into one blastomere on the animal pole at the 128-cell stage and then detected at 6 hpf. **(I)** ChIP-qPCR analysis of Junb binding to upstream regions of dhrs9. Embryos injected with HA-Junba mRNA were subjected to chromatin immunoprecipitation with HA-agarose, using IgG-agarose as a control. Enrichment at three predicted binding sites upstream of the dhrs9 gene was quantified by qPCR. Data are presented as the mean ± SD from three independent biological replicates. ***P < 0.001 (Unpaired *t t*est). **(J** and **K)** WISH of *spi1b* in *angptl5*^*Δ10/Δ10*^ embryos. Embryos were injected with *itga6l + itgb5* mRNA at the 1-cell stage and treated with or without ERK inhibitor from the shield stage to the 18-somite stage (J), or injected with *caERK*2 mRNA at the 1-cell stage and treated with or without RA receptor antagonist AGN 193109 (K). Uninjected embryos were used as control. Statistics are shown below. Data presented as the mean ± SD, n(*angptl5*^*Δ10/Δ10*^) = 22, n(*angptl5*^*Δ10/Δ10*^ + *itga6l* + *itgb5*) = 26, n(*angptl5*^*Δ10/Δ10*^ + *itga6l* + *itgb5* + ERKi) = 28 (J); n(*angptl5*^*Δ10/Δ10*^) = 22, n(*angptl5*^*Δ10/Δ10*^ + *caERK*) = 22, and n(*angptl5*^*Δ10/Δ10*^ + *caERKi* + AGN) = 26 (K). Statistical significance: **P < 0.01, ***P < 0.001 (One-way ANOVA). LV, lateral view; AV, animal view (C–H), anterior view (J and K). The data for this figure can be found in S1 Data.

Building on our previous characterization of RA signaling in Angptl5-dependent hematopoiesis, we investigated whether the Angptl5/Integrin α6lβ5-ERK axis can directly modulates RA activity. Using *cyp26a1* as a readout of RA pathway activation, we found that ERK inhibition significantly reduced the *cyp26a1* expression in the WT embryos, while ERK activation drastically enhanced *cyp26a1* expression. Interestingly, triple overexpression of *angptl5*, *itga6l*, and *itgb5* at one cell stage also dramatically increase the *cyp26a1* expression. Conversely, CRISPR-Cas9-mediated knockdown of *itga6l* and *itgb5* significantly reduced *cyp26a1* expression (S13A Fig). Importantly, this Angptl5/Integrin-mediated *cyp26a1* induction was completely abolished by ERK inhibition, demonstrating that the Angptl5/integrin complex enhances RA signaling through ERK-dependent mechanisms (Fig 6A).

To further explore the mechanism by which the Angptl5/Integrin α6lβ5-ERK axis regulates RA activity, we injected *caERK2* mRNA or *angptl5*, *itga6l*, and *itgb5* mRNA together into one blastomere at the 128-cell stage, followed by analysis of the expression of RA synthases *aldh1a2* and *dhrs9* (Fig 6B). Interestingly, both treatments induced *dhrs9* expression at the animal pole, but not *aldh1a2* (Fig 6C and 6D). To systematically dissect the activating effects of these components on *dhrs9* transcription, we performed a two-stage microinjection in zebrafish embryos (Fig 6E). Our results showed neither *angptl5* nor *itga6l* was sufficient to induce *dhrs9* expression. Intriguingly, *itgb5* alone, or together with *angptl5*, caused a slight induction of *dhrs9* expression. Co-injection of *itga6l* and *itgb5* promoted a moderate transcriptional activation of *dhrs9*, and this activation was dramatically boosted by the additional overexpression of *angptl5*. Critically, this synergistic activation was completely abolished upon pharmacological ERK inhibition (Fig 6F and 6G), suggesting its dependence on ERK signaling.

To determine the specific upstream kinase responsible of ERK activation [41], we performed a targeted inhibitor screen. Our results revealed that ERK activation downstream of the Angptl5-Integrin α6lβ5 complex is dependent on Src family kinase activity, as it was abolished by Src inhibition (S12F Fig). In parallel, we also tested two alternative mechanisms: first, whether the complex signals through the canonical integrin effector FAK; and second, whether it facilitates ERK activation indirectly by enhancing EGFR signaling. Inhibition of either FAK or EGFR had no effect on Angptl5/Integrin α6lβ5 mediated ERK activation (S12B and S12E Fig), indicating that neither protein was required for the regulatory axis.

Finally, to investigate how ERK signaling activates *dhrs9* transcription, we performed a bioinformatic analysis of the *dhrs9* promoter region, which revealed three conserved, high-probability binding sites for the transcription factor Junb—a canonical nuclear target of the ERK/MAPK pathway [42]—predicted based on UCSC Genome Browser and JASPAR database analyses. Knockdown of *junba/junbb* completely abolished the ectopic induction of *dhrs9* by caERK2 (Fig 6H). Chromatin immunoprecipitation (ChIP) assays further confirmed the specific enrichment of Junb at these predicted sites in vivo (Fig 6I).

In summary, these findings indicate a complete signaling transduction cascade: Angptl5, together with Itga6l/Itgb5, activates a Src-dependent ERK pathway, resulting in Junb-dependent transcriptional activation of *dhrs9*, thereby modulating RA activity. This model was further supported by the observation that the RA signaling potentiation caused by co-overexpression of *angptl5*, *itga6l,* and *itgb5* in *angptl5*^*Δ10/Δ10*^ embryos was remarkedly attenuated by ERK inhibition (S14A Fig).

Next, we validated the regulatory role of the Angptl5/Integrin α6lβ5-ERK-Junb-RA signaling axis in primitive hematopoiesis. Co-injection of *itga6l* and *itgb5* mRNA into *angptl5*^*Δ10/Δ10*^ embryos partially attenuated myeloid hyperplasia, but this suppression was reversed upon ERK inhibitor treatment, revealing that the regulatory function of integrin α6lβ5 in primitive hematopoiesis is mechanistically reliant on ERK signaling activation (Fig 6J). In contrast, *caERK2* overexpression completely abolished *spi1b* expression in *angptl5*^*Δ10/Δ10*^ embryos, but this inhibitory effect was counteracted by RA inhibitor treatment, suggesting that ERK signaling-mediated regulation of primitive hematopoiesis requires RA activity (Fig 6K).

Taken together, these findings establish Angptl5 as a critical regulator of zebrafish primitive hematopoiesis, operating through the Angptl5/Integrin α6lβ5-ERK-Junb-RA signaling axis to suppress excessive expansion of hematopoietic progenitor cells.

## Discussion

**Fig 7:**
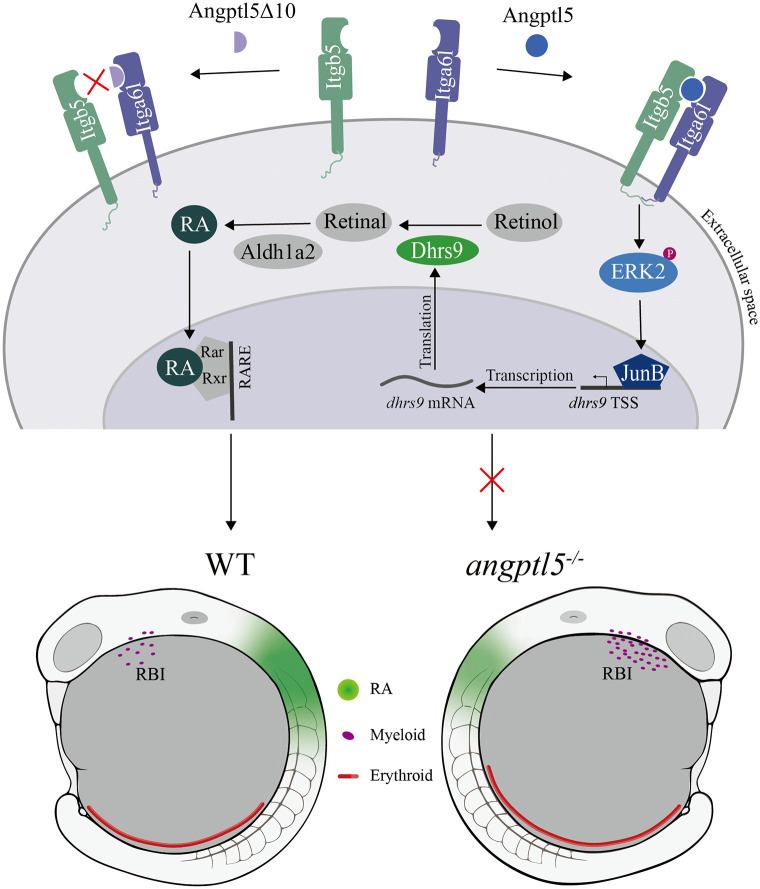
Working model of Angptl5-mediated regulation of primitive hematopoiesis in zebrafish. During primitive hematopoiesis, Angptl5 (but not the truncated Angptl5Δ10) specifically promotes the assembly of Itga6l and Itgb5 subunits into functional integrin α6lβ5 heterodimers, thereby activating downstream ERK signaling pathways. Activated ERK2 then induces *dhrs9* expression via the transcription factor Junb, which binds directly to the *dhrs9* promoter. This upregulation of *dhrs9* potentiates RA signaling, ultimately suppressing excessive expansion of myeloid and erythroid progenitors in primary hematopoiesis.

Our study establishes Angptl5 as a novel and essential regulator of primitive hematopoiesis in zebrafish. We define a linear Angptl5-Integrin α6lβ5-ERK-RA signaling axis that controls this process. Specifically, we demonstrate that Angptl5 binds to and promotes the assembly of the Integrin α6lβ5 heterodimer, thereby activating downstream ERK signaling. The activated ERK pathway, in turn, upregulates the RA synthase gene *dhrs9* through its canonical transcription factor target Junb, ultimately enhancing RA biosynthetic activity. Thus, this previously unrecognized regulatory axis functions as a critical developmental brake to suppress the excessive expansion of both myeloid and erythroid progenitor populations during zebrafish embryogenesis (summarized in Fig 7).

Members of the Angiopoietin-like (Angptl) protein family play crucial roles in various physiological and pathological processes, including metabolism [43,44], inflammation [13,45], and cancer [46]. Several members of this family, including Angptl5, have been reported to *ex vivo* positively regulate the maintenance and proliferation of stem cells, including HSCs [18,19,47]. However, in our study, we observed that the loss of Angptl5 did not lead to significant changes in primitive HSCs. This may be due to the redundancy or compensatory mechanisms provided by other Angptl family members in vivo.

Interestingly, the loss of Angptl5 led to the simultaneous expansion of myeloid cells at the anterior of the body axis and erythroid cells at the posterior, despite their established mutual antagonism [48,49]. This suggests that the hematopoietic microenvironment plays a specific and critical role in maintaining the proliferative capacity of hematopoietic progenitors at different stages. This also raises an important question: despite both anterior and posterior hematopoietic progenitors originating from the same primitive HSC [50], what signals in the anterior and posterior microenvironments induce their distinct differentiation into myeloid and erythroid progenitors, respectively? How does the Angptl5-RA axis interact with these key signals? These questions provide an exciting avenue for future research. Although, this study defines the developmental origin and molecular mechanism driving the hematopoietic expansion in *angptl5* mutants, the functional consequences of this increased neutrophil and macrophage population—such as its impact on immune response or homeostasis—remain an open and intriguing question for future investigation.

Our mechanistic studies identify ERK as a central hub in Angptl5-dependent hematopoietic control, promoting RA synthesis through transcriptional activation of dhrs9. Notably, this forms a potential feedback loop, as previous studies show that RA can rapidly activate ERK via noncanonical, receptor-independent mechanisms [51,52]. This bidirectional interaction between RA and ERK suggests a tightly regulated circuit with possible roles in fine-tuning hematopoietic processes. Further investigation is warranted to explore how this circuit functions across developmental stages, tissues, and in both normal and disease contexts.

In summary, we identify Angptl5 as a critical rheostat of primitive hematopoiesis, bridging integrin-mediated signaling (Itga6l/Itgb5) with RA metabolic regulation (Dhrs9). This axis ensures precise control of myeloid-erythroid progenitor expansion during embryogenesis, with perturbations leading to hematopoietic hyperplasia. Notably, a murine ortholog of ANGPTL5 has not been identified to date, making zebrafish a particularly relevant model for studying its developmental functions. While this precludes direct mammalian genetic validation, it also highlights the unique evolutionary niche in which this signaling axis operates. These findings not only expand the known functions of ANGPTL family proteins beyond angiogenesis and metabolism, but also elucidate an intricate signaling cascade that coordinates hematopoietic homeostasis in the embryo. More broadly, this work reveals a novel developmental paradigm in which a secreted extracellular signal (Angptl5) is perceived by a specific integrin receptor (α6lβ5) to modulate RA gradients. Furthermore, the Angptl5-integrin-ERK axis may represent a potential therapeutic target for disorders driven by RA-ERK imbalance, such as chemotherapy-resistant acute myeloid leukemia (AML) [53].

## Materials and methods

### Ethics statement

All fish maintenance and experimental procedures were performed in strict accordance with the Regulations for the Administration of Affairs Concerning Experimental Animals and the National Standard of the People’s Republic of China for the Care and Use of Laboratory Animals (GB/T 35892-2018). The study protocol was approved by the Animal Care and Use Committee of Zhejiang University (Protocol No. ZJU20220375).

### Zebrafish husbandry and genetic strains

Zebrafish were raised on a 14- hours-light/10-hours-dark cycle at 28 °C [54] and staged as described [55]. The following lines were used in this study: wild-type (WT) AB strains, *Tg(angptl5:EGFP), Tg(RARE:EGFP)*, *Tg(kdrl:GFP)*, *angptl5*^*Δ10/Δ10*,^ and *angptl5*^*Δ5/Δ5*^ mutants. All fish maintenance was performed per the requirements of the “Regulation for the Use of Experimental Animals in Zhejiang Province”, with the approval by the Zhejiang University Animal Care and Use Committee. The transgenic line *Tg(spi1b:EGFP)* [56] was obtained from Shanghai Institute of Hematology at Ruijin Hospital, SJTU.

### WISH and histological sections

WISH of zebrafish was performed essentially as described previously [57]. Probe templates including *tal1*, *spi1b*, *gata1a*, *mpx*, *lyz*, *mpeg1.1*, *myod1*, *aldh1a2*, *dhrs9*, *cyp26a1*, *angptl1–7*, *etsrp*, *fli1,* and *kdrl* have been generated from zebrafish cDNA and clones inserted into the pEASY-blunt-zero vector (Transgen). Antisense RNA probes were labeled with DIG RNA labeling Mix (Roche) by transcription using T7 or T3 polymerase (Promega) in vitro.

For histological sections, embryos were washed with PBS following WISH and embedded in OCT compound (optimal cutting temperature compound, Sakura). Cryosections of 20 μm thickness were prepared along the anterior–posterior axis using an NX50 Cryostat Microtome (Thermo Scientific), and images were captured with an ECLIPSE Ni microscope.

### Generation of Tg(*angptl5*:EGFP) and Tg(*RARE*:EGFP) transgenic lines

The *angptl5:EGFP* plasmid was generated by cloning the *angptl5* promoter by cloning the upstream 867 bp region of *angptl5* gene and EGFP fragment into the Tol2 backbone using a seamless cloning kit (Vazyme). For the *RARE:EGFP* plasmid, the EF1α enhancer-promoter in the PT2AL200R150G transposon vector [58] was replaced with a trimerized *RARE* sequence, which was amplified from the pGL3-RARE-luciferase plasmid (Addgene). Both plasmids were co-injected with *Tol2* mRNA into zebrafish embryos at the 1-cell stage to generate the *Tg(angptl5:EGFP)* and *Tg(RARE:EGFP)* transgenic lines, respectively.

### In situ hybridization chain reaction (HCR)

HCR staining was performed as described previously [59]. Briefly, zebrafish embryos were fixed with 4% (w/v) paraformaldehyde in DEPC-treated PBS at 4 °C overnight. Samples underwent proteinase K (MCE) treatment before incubation of probe. Samples were incubated with 2 pmol of each HCR probe sets in 100 μL probe hybridization buffer at 37 °C overnight. Hybridization was terminated by washing samples repeatedly with probe wash buffer at room temperature. For amplification, the probes were conjugated with fluorescent HCR amplifiers in amplification buffer for 4 hours at room temperature in the dark. The reaction was stopped by washing several times with 5× SSCT (DAPI co-staining was achieved by adding 0.1 μg/mL DAPI). Fluorescent hairpins, buffers, and DNA probes were purchased from Molecular Technologies. Fluorescent signals were detected using an OLYMPUS CSU-W1 confocal microscope.

### Generation of *angptl5*^*Δ10/Δ10*^ and *angptl5*^*Δ5/Δ5*^ mutants using CRISPR/Cas9

To generate the *angptl5*^*Δ10/Δ10*^ and *angptl5*^*Δ5/Δ5*^ mutants, we synthesized gRNAs targeting the fourth exon and third exon of the zebrafish *angptl5* gene, respectively. These procedures were performed as previously described [60]. The guide RNAs (S1 Table), targeting zebrafish *angptl5* gene, were designed using CHOPCHOP (http://chopchop.cbu.uib.no) and synthesized using the T7 High Yield RNA Transcription Kit (Vazyme). The SpyCas9 protein (NEB) and *angptl5*-targeting gRNA were co-injected into WT embryos at the 1-cell stage. The *angptl5* mutant lines were identified in the F1 generation by analyzing the PCR products using the primer pair listed in S1 Table.

### Immunofluorescence

Whole-mount immunofluorescence staining was performed as described previously [61] using the following primary antibody: Phospho-Erk1 (Thr202/Tyr204)/Erk2 (Thr185/Tyr187) rabbit monoclonal antibody (Beyotime, 1:100), and Alexa Fluor 488-conjugated donkey anti-rabbit antibody (Invitrogen, 1:500 dilution) used as the secondary antibody. Embryos were photographed using an OLYMPUS CSU-W1 confocal microscope.

### Preparing samples for sequencing and pro-processing single cell RNA-seq data

The *angptl5*^*Δ10/Δ10*^ embryos were raised in 0.3× Danieau Buffer and harvested at 16 hpf. Collected samples were digested with trypsin into single cell suspension and set to Novogene for single cell sequencing.

### Pro-processing single cell RNA-seq data

The raw Illumina sequencing reads were aligned to the zebrafish reference genome (GRCz11) through 10×  Genomics CellRanger pipeline v3.0.2 with default parameters. The *angptl5*^*Δ10/Δ10*^ embryos yielded 7,350 cells, and the generated expression matrix was further processed by Seurat v5.1.0 [62]. Low-quality cells with fewer than 200 detected genes, more than 7,000 detected molecules, or greater than 15% mitochondrial expression were filtered out. The single cell RNA-seq dataset of 16 hpf WT embryos was obtained from the published work [21]. The single cell RNA-seq datasets of WT and *angptl5*^*Δ10/Δ10*^ mutant embryos were merged for further analyses. The NormalizeData function with default settings was used to normalized the raw counts. The FindVariableFeatures function was used to identify the highly variable genes. The normalized data was then scaled with the through ScaleData function. Principle component analysis (PCA) and UMAP were employed to reduce the dimensionality of the data.

### Integrative single cell RNA-seq analysis

The merged datasets were integrated using IntegrateLayers function with the canonical correlation analysis (CCA) integration method, utilizing shared principal components across two conditions. The layers of two conditions were re-joined after integration through the JoinLayers function. Spot clusters were obtained using the FindNeighbors and FindClusters function with the first 30 dimensions and the resolution set to 0.8. Nonlinear reduction was run using RunUMAP function with the first 30 dimensions. Marker genes for each cluster were identified using the FindAllMarkers function, and each cluster was then annotated based on the expression of these marker genes. The WT embryos had a total of 11,460 cells, with 170 erythroid progenitor cells (1.483%) and 19 myeloid progenitor cells (0.165%); while the *angptl5*^*Δ10/Δ10*^ mutant embryos contained 7,241 total cells, with 192 erythroid progenitor cells (2.652%) and 32 myeloid progenitor cells (0.442%).

### Analysis of differential abundance in cell types

Milo [63] was employed to analyze the differential abundance in cell types between WT and *angptl5*^*Δ10/Δ10*^ embryos. Briefly, a k-nearest neighbor (KNN) graph was firstly constructed, and cells were assigned to the defined representative neighborhoods. The cells belonging to WT and *angptl5*^*Δ10/Δ10*^ embryos in each neighborhood were counted. Differential abundance analysis was conducted within neighborhoods using the negative Binomial generalized linear model (GLM), with p-values corrected using the Spatial FDR method. A cell type label was assigned to each neighborhood by identifying the most abundant cell type among the cells within that neighborhood. A beeswarm plot was used to visualize the distribution of differential abundance fold changes among different cell types.

### Chemical treatment

In general, embryos were treated with drugs from the shield stage until collection at the indicated stages. When injected at the 128-cell stage, embryos received drug treatment immediately post-injection with continuous exposure until specimen collection at the shield stage.

The drug was initially dissolved in DMSO to prepare a high-concentration stock solution, which was subsequently diluted with 0.3×Danieau buffer to the working concentration. Detailed pharmacological parameters and experimental concentrations are tabulated below: RA (sigma), Aldehyde dehydrogenase inhibitors 4-diethylaminobenzaldehyde (DEAB) (MCE, 10 μM), RA receptor (RARs) antagonists AGN 193109 (MCE, 20 μM), FAK inhibitor Defactinib (MCE, 2 μM/8 μM), MAPK inhibitor Adezmapimod (MCE, 10 μM), ERK inhibitor Mirdametinib (MCE, 10 μM), ERK activator C16-PAF (MCE, 1 μM/5 μM), NF-κB inhibitor BAY 11-7082 (MCE, 0.1 μg/mL, 0.4 μg/mL).

### Quantitative Real-time PCR

Total RNA was isolated from zebrafish embryos (50 embryos per sample) using RNA isolater Total RNA Extraction Reagent (Vazyme), followed by cDNA synthesis with HiScript III 1st Strand cDNA Synthesis Kit (+gDNA wiper) (Vazyme). qPCR was conducted on a LightCycler 480 Instrument II system (Roche Diagnostics) with 2× Universal SYBR Green Fast qPCR Mix (ABclonal). The ΔΔCT method employed for data analysis, with normalization to 18S ribosomal RNA (18s rRNA) levels. Primer sequences with annealing temperatures are detailed in S1 Table.

### Gene knockdown by morpholino and CRISPR/Cas9

For transient gene knockdown, two approaches were employed. Morpholino antisense oligonucleotides (Gene Tools LLC) were used to inhibit *itga6l* (5′- GCTCTCCTTTCTTCATCAGGTTCAT-3′) and *shc1* (5′- ATAAAGAATTGGAAACCTTTCTCCT-3′). Embryos at the one-cell stage were microinjected with 60 nM (*itga6l*) or 3 nM (*shc1*) morpholino solution using standard procedures.

Alternatively, transient CRISPR/Cas9-mediated knockdown was performed for *itga6l* and *itgb5*. Gene-specific guide RNAs (gRNAs, sequences listed in S1 Table) were synthesized in vitro using the T7 High Yield RNA Transcription Kit (Vazyme). A mixture of each gRNA and SpyCas9 protein (NEB) was co-injected into WT embryos at the one-cell stage. This method achieves efficient somatic mutation and gene disruption for phenotypic analysis within the injected generation (F0).

### Plasmid constructs

The full-length *angptl5* coding sequence was amplified and cloned into a pCS2(+) vector with the addition of Flag or Flag-TurboID tag in frame at the C terminus. Based on Flag or Flag-TurboID tagged Angptl5 plasmid, Angptl5Δ10 was constructed by deleting 10 bp (base pair 458–467). Angptl5 and Angptl5Δ10 tagged with Flag or V5 were used for Immunoblotting (IB) and co-immunoprecipitation (Co-IP) in zebrafish embryos. Angptl5 and Angptl5Δ10 tagged with Flag-TurboID were used for IP-MS in zebrafish embryos. For Co-IP analysis in zebrafish embryos, the *itga6l* and *itgb5* coding sequences were amplified and cloned into a pCS2(+) vector, with addition of HA, V5, or Flag tag in frame at the C terminus.

### Co-IP and mass spectrometry

Co-IP analysis in zebrafish embryos was performed as described previously [64]. Briefly, embryos were co-injected with the indicated mRNA as described in the main text at the 1-cell stage, and collected at 5–6 hpf. Then, embryos were lysed with ice-cold lysis buffer [50 mM Tris at pH 7.5, 150 mM NaCl, 10% glycerol, 1% Triton X-100, and complete protease inhibitor (Roche)]. Lysates were centrifuged and the supernatants were then transferred to a Spin Column (Pierce). Samples were incubated with HA-Nanoab-Agarose (LabLEAD), Anti-Flag Affinity Gel (Bimake), or DYKDDDDK-Nanoab-Agarose (LabLEAD) at 4 °C overnight, followed by four washes with ice-cold wash buffer (50 mM Tris at pH 7.5, 150 mM NaCl, 1%Triton X-100 and complete protease inhibitor) four times before adding IgG Elution Buffer (Pierce).

IP-MS was performed as described [32]. Briefly, 800 embryos were lysed and subjected to immunoprecipitation with Streptavidin Agarose Resins (Pierce). Samples were separated by SDS-PAGE. The excised gels were analyzed by liquid chromatography-tandem mass spectrometry (LC-MS/MS) at Shanghai Applied Protein Technology Co., Ltd (APTBIO, China).

### Chromatin immunoprecipitation and qPCR (ChIP-qPCR)

To assess the binding of Junb to the upstream region of the *dhrs9* gene, ~300 zebrafish embryos were injected at the one-cell stage with HA-Junba mRNA and collected at 6 hpf. Following fixation with 1.45% formaldehyde, embryos were deyolked and lysed, and the lysate was treated with RNase A prior to chromatin shearing by sonication. Prior to immunoprecipitation, 10% of the sample was reserved as input. Chromatin was incubated with HA-agarose or control IgG-agarose beads overnight at 4 °C. Following sequential washes, bound complexes were eluted, reverse-crosslinked, and the DNA was purified. Quantitative PCR was performed to measure enrichment at three putative Junb binding sites upstream of *dhrs9*, predicted by analyses using the UCSC Genome Browser and the JASPAR database. Data were normalized to the input control.

### Western blot analysis

Western blot analysis was performed as described previously [65]. Briefly, embryos or HEK293T cells were lysed with ice-cold RIPA Complete Lysis Buffer (Beyotime). For HEK293T conditioned medium (CM) preparation, the plasmids were transfected using polyethylenimine (PEI, Polysciences) at 1:2.5 DNA ratio. At 24 hours post-transfection, medium was replaced with serum-free DMEM. After 24 hours of incubation, CM samples were collected, centrifuged, and concentrated 5–10-fold using centrifugal filters (Millipore). Proteins were subjected and separated on SDS‐PAGE gel and transferred to PVDF membranes (Millipore). After blocking with nonfat milk (CST), membranes were incubated overnight with the following specific primary antibodies: ERK1/2 (Beyotime), Phospho-Erk1 (Thr202/Tyr204)/Erk2 (Thr185/Tyr187) (Beyotime), Beta Tubulin (Proteintech), Flag-Tag (Beyotime), HA-Tag (CST), and V5-Tag (Invitrogen). After four TBST washes, membranes were incubated with appropriate HRP‐conjugated secondary antibodies. After incubation, membranes were subjected to five TBST washes, then visualized using BeyoECL Moon Kit (Beyotime) or BeyoECL Star Kit (Beyotime) and detected with gel image analysis system (Tanon).

### Statistical analysis

Raw image data were processed using ImageJ software. Normally distributed data were presented as mean ± SD. Comparisons between two groups were performed using the unpaired two-tailed Student *t* test, while comparisons among three or more groups were performed using one-way ANOVA followed by Tukey’s post hoc test. A *p*-value of less than 0.05 was considered statistically significant. The statistical parameters were shown in the figures and described in the figure legends or in the main text. All statistical analyses were performed using GraphPad Prism 9.5 (GraphPad Software, USA).

## Supporting information

S1 Fig Cross-species evolutionary comparison of ANGPTL5. (**A**) Phylogenetic tree of *ANGPTL5* nucleotide sequence in human, rabbit, chick, *Xenopus* and zebrafish using the neighbor joining method. (**B**) ANGPTL5 protein sequence homology among different species. (**C**) Structural models of the zebrafish and human ANGPTL5 proteins were modeled using AlphaFold3. CCD, coiled-coil domain; FLD, fibrinogen-like domain.
(TIF)

S2 Fig Expression patterns of *angptl* family members during zebrafish embryonic development. (**A**) WISH of *angptl* family members in zebrafish embryos. (**B**) WISH of *angptl5* during zebrafish embryonic development at the indicated stages. (**C**) Cryosection of the trunk region for 24 hpf zebrafish embryo using O.C.T. after WISH of *angptl5*. (**D**) Time-lapse confocal micrographs of the *angptl5* positive cell in *Tg(angptl5:*EGFP) embryos. Each imaging was performed for at least three independent replicates. LV, lateral view; DV, dorsal view; AV, anterior view. NT, Neural tube; NC, notochord; ICM, intermediate cell mass.
(TIF)

S3 Fig Comparison of primitive hematopoiesis between WT and *angptl5*^*Δ10/Δ10*^ embryos. (**A**) Schematic diagram showing the positions of gRNA target site; the deletions of *angptl5* mutant lines generated using CRISPR/Cas9; and the predicted truncated Angptl5Δ10 and Angptl5Δ5 proteins (predicted domains taken from the Uniprot database). (**B**) WISH of *tal1* in WT and *angptl5*^*Δ10/Δ10*^ embryos. (**C**) WISH of neutrophil markers *lyz, mpx* and macrophage marker *mpeg1.1* in WT and *angpt15*^*Δ10/Δ10*^ embryos. (**D** and **E**) WISH of *spi1b* (D) and *lyz* (E) in WT and *angptl5*^*Δ5/Δ5*^ embryos. (**F**) qPCR shows the *tal1*, *spi1b*, *gata1a* and *etsrp* expression in WT and *angptl5*^*Δ10/Δ10*^ embryos. Data presented as the mean ± SD, *n* = 30. Three independent biological replicates were used. Statistical significance: ***P < 0.001 (Unpaired *t* test). (G) Comparison of survival rates among WT, *angptl5*^*Δ10/Δ10,*^ and *angptl5*^*Δ5/Δ5*^ zebrafish. (Kaplan–Meier survival curves of WT (blue solid line), *angptl5*^*Δ10/Δ10*^ (red solid line), and *angptl5*^*Δ5/Δ5*^ (green solid line) zebrafish under normal rearing conditions up to 20 days post-fertilization (dpf). The survival curves were compared using the Log-rank (Mantel–Cox) test (*P* = 0.7982, not significant (ns). Each genotype group consisted of *n* = 100). LV, lateral view; AV, anterior view. The data for this figure can be found in S1 Data.
(TIF)

S4 Fig Integrative single cell RNA-seq analysis of 16 hpf WT and *angptl5*^*Δ10/Δ10*^ embryos. (**A**) Integrative UMAP analysis of 16 hpf zebrafish embryos, with the UMAP results separated by the conditions of WT and *angptl5*^*Δ10/Δ10*^ embryos. The WT dataset is from the published work [21]. Each cell is coloured according to cell type annotations. (**B**) Dot plot showing the expression of two representative markers for each cluster. Color represents the gene expression level, while dot size indicates the percentage of cells within the cluster expression the gene.
(TIF)

S5 Fig Comparison of primitive hematopoiesis and angiogenesis between WT and *angptl5*^*Δ10/Δ10*^ embryos. (**A**–**C**) WISH of *etsrp* (A), *fli1* (B) and *kdrl* (C) in WT and *angptl5*^*Δ10/Δ10*^ embryos. (**D**) Intersegmental blood vessels in WT and *angptl5*^*Δ10/Δ10*^ embryos shown by *fli1:GFP* reporter. Each imaging was performed for at least three independent replicates. AV, anterior view; LV, lateral view; DV, dorsal view.
(TIF)

S6 Fig Retinoic acid signaling in early zebrafish development. (**A**) WISH of *fn1a* in WT and *angptl5*^*Δ10/Δ10*^ embryos. (**B**) Schematic diagram of RA signaling pathway. (**C**) RA signaling in *Tg(RARE:EGFP)* embryos treated with RA from the shield stage to 24 hpf. Untreated embryos were used as control. (**D**) Morphology of WT embryos treated with RA inhibitor DEAB or different RA concentrations from the shield stage to 18-somite stage. Untreated embryos were used as control. (**E**) WISH of *cyp26a1* in WT embryos treated with DEAB or RA from the shield stage to 8 hpf. Untreated embryos were used as control. LV, lateral view.
(TIF)

S7 Fig Regulation of *spi1b* and *gata1a* expression by Retinoic Acid Signaling. (**A** and **B**) WISH of *spi1b* in WT embryos treated with RA (A) or RA inhibitor AGN (B). Untreated embryos were used as control. (**C**) WISH of *gata1a* and *myod1* in flat-mounted WT and *angptl5*^*Δ10/Δ10*^ embryos treated with RA inhibitor DEAB or AGN. (**D**) WISH of *cyp26a1* in WT and *angptl5*^*Δ10/Δ10*^ embryos injected with *dhrs9* or *aldh1a2* mRNA at the 1-cell stage. Uninjected embryos were used as control. Statistics are shown on the right of the representative photos. AV, anterior view; LV, lateral view. The data for this figure can be found in S1 Data.
(TIF)

S8 Fig Secretion and functional analysis of tagged Angptl5. (**A**) Synthesis and secretion analysis of Angptl5-Flag-TurboID and Angptl5Δ10-Flag-TurboID in HEK293T cells after transfection with Angptl5-Flag-TurboID and Angptl5Δ10-Flag-TurboID plasmid for 48 hours. Conditioned medium (CM) were collected for western blot. (**B**) Expression of Angptl5-Flag-TurboID, Angptl5Δ10-Flag-TurboID and biotinylated proteins in zebrafish embryos. Embryos were injected with *Angptl5-Flag-TurboID* and *Angptl5Δ10-Flag-TurboID* mRNA at the 1-cell stage, and then embryos were collected for western blot at the 75% epiboly. (**C**) WISH of *spi1b* in *angptl5*^*Δ10/Δ10*^ embryos injected with *angptl5*, *angptl5-flag,* or *angptl5-TurboID* mRNA at the 1-cell stage. Uninjected embryos were used as control. (**D**) WISH of *spi1b* in *angptl5*^*Δ10/Δ10*^ embryos. Embryos were injected with *angptl5* mRNA ± *itga6l* MO at the 1-cell stage. Uninjected embryos were used as control. AV, anterior view.
(TIF)

S9 Fig Interaction between Itga6l and integrin β-subunits. (**A**) Expression level of integrin β-subunits in zebrafish embryos from published work [35]. (**B–F**) Structural models of the extracellular domain complex between Itga6l and: Itgb1a (B); Itgb1b (C); Itgb1b.1 (D); Itgb6 (E); Itgb4 (F). (**G**) Structural models of the Angptl5_78–397_-Itga6l_14-736_-Itgb4_25–710_ complexes. (**H** and **I**), WISH of *itga6l* (H) and *itgb5* (I) in WT embryos. The three-dimensional structures of all protein complexes were predicted using AlphaFold3 via the AlphaFold Server (https://alphafoldserver.com). The top-ranked model based on the predicted template modeling score was selected for further analysis. All structural figures were generated using the open-source version of PyMOL. DV, dorsal view; LV, lateral view; AV, anterior view.
(TIF)

S10 Fig Analysis of integrin α6lβ5 in regulating primitive hematopoiesis. (**A**) WISH of *spi1b*, *gata1a,* and *myod1* in WT embryos treated at the 1-cell stage as follows: injection of Cas9/gRNA or treatment with Integrin inhibitor (Cilengitide) from 6 hpf. KD: knockdown; (**B**) WISH of *spi1b* in WT embryos injection of respective mRNAs. Uninjected embryos were used as control. (**C** and **D**) WISH of *spi1b* (C), *gata1a* and *myod1* (D) in WT embryos with *angptl5 + itga6l + itgb5* mRNA at the 1-cell stage at the 1-cell stage. AV, anterior view.
(TIF)

S11 Fig ERK signaling is reduced in *angptl5*^*Δ10/Δ10*^ embryos. (**A–C**) WISH of *spi1b or cyp26a1* in WT embryos treated with FAK inhibitor (A), NF-κB inhibitor (B), MAPK inhibitor and ERK inhibitor (C) from shield stage to 18-somite stage. Untreated embryos were used as control. (**D**) Schematic diagram of ERK2 and constitutively activated ERK2 (caERK2). (**E**) WISH of *spi1b* in WT embryos injected with *ERK2* or *caERK2* mRNA at the 1-cell stage. Uninjected embryos were used as control. (**F**) Projection of images from confocal stacks to show co-localization of *spi1b* and p-ERK in *Tg(spi1b:EGFP)*. Each imaging was performed for at least three independent replicates. (**G**) Cryosection of the trunk region for 24 hpf WT zebrafish embryo using O.C.T. after IF of p-ERK. NT, Neural tube; ICM, intermediate cell mass. (**H**) Projection of trunk region images from confocal stacks to show p-ERK1/2 in WT and *angptl5*^*Δ10/Δ10*^ embryos by IF. (**I**) Expression of p-ERK in WT embryos injected with *angptl5* + *itga6l* + *itgb5* mRNA at the 1-cell stage. Statistics are shown on the right. AV, anterior view; LV, lateral view. The data for this figure can be found in S1 Data.
(TIF)

S12 Fig Kinases and adaptor proteins mediating Angptl5-Itga6lβ5-induced ERK signaling identified by immunofluorescence. (**A–F**) IF of p-ERK1/2 in WT embryos. Embryos were injected in one animal pole blastomere at the 128-cell stage with the indicated mRNAs or MO, and then treated as follows: with the FAK inhibitor Defactinib (B); with the EGFR inhibitor Gefitinib (D and E); with the Src inhibitor Saracatinib (F); or with a *shc1* MO (**G** and **H**). All embryos were imaged at 6 hpf. AV, animal view.
(TIF)

S13 Fig Integrin α6lβ5 regulates RA signaling. (**A**) WISH of *cyp26a1* in WT embryos. Embryos were co-injected with Cas9 protein and gRNAs target against *itga6l* and *itgb5* at the 1-cell stage. Untreated embryos were used as control. KD: knockdown; LV, lateral view.
(TIF)

S14 Fig RA signaling is regulated by Angptl5-Integrin α6lβ5-ERK signaling cascade. (**A**) RA signaling in *angptl5*^*Δ10/Δ10*^ embryos injected with *angptl5* + *itga6l* + *itgb5* mRNA at the 1-cell stage and treated with or without ERK inhibitor from the shield stage to 18-somite stage. Data presented as the mean ± SD, n(*angptl5*^*Δ10/Δ10*^) = 19, n(*angptl5*^*Δ10/Δ10*^ + *angptl5* + *itga6l* + *itgb5*) = 20, n(*angptl5*^*Δ10/Δ10*^ + *angptl5* + *itga6l* + *itgb5 + ERKi*) = 16. Statistical significance: ***P < 0.001 (One-way ANOVA). The data for this figure can be found in S1 Data.
(TIF)

S1 Table The primers used in this work including gene knockout, qPCR, and CHIP-qPCR. (PDF)

S1 Data Underlying numerical data for all main and supporting figures. (XLSX)

S1 Dataset Candidate Angptl5-associated proteins identified by IP-MS related to Fig 4B. (XLSX)

S1 Raw Images Raw images of Figs 4D and 4F, 4I, 5E, S8A and S11I. (PDF)

## Abbreviations

ALPM: anterior lateral plate mesoderm
AML: acute myeloid leukemia
Angptl5: angiopoietin-like protein 5
ANGPTLs: angiopoietin-like proteins
CCA: canonical correlation analysis
CCD: coiled-coil domain
ChIP: chromatin immunoprecipitation
CM: conditioned medium
FLD: fibrinogen-like domain
GFP: green fluorescent protein
GLM: generalized linear model
HCR: hybridization chain reaction
hpf: hours post-fertilization
HSCs: hematopoietic stem cells
ICM: intermediate cell mass
IF: immunofluorescence
IP-MS: immunoprecipitation-mass spectrometry
Itga6l: integrin α6-like
LDO: Laboratory of Development and Organogenesis
PCA: principle component analysis
pERK1/2: phosphorylated ERK1/2
PLPM: posterior lateral plate mesoderm
qPCR: quantitative real-time PCR
RA: retinoic acid
RARs: retinoic acid receptor
RBI: rostral blood island
scRNA-seq: single-cell RNA sequencing
WISH: whole-mount in situ hybridization
WT: wild-type.
