# Supplementary material for: Angptl5 restricts primitive hematopoiesis by promoting retinoic acid signaling in zebrafish
Source: PLoS Biol. 2026 Jun 25;24(6):e3003858. doi: 10.1371/journal.pbio.3003858 (PMC13298773; doi:10.1371/journal.pbio.3003858)
Supplement: S1 Table — (PDF) [file pbio.3003858.s015.pdf]

Primer Name	Application	Target gene	Sequence 5'- 3'
A5_E4_gRNA	gene knockout	*angptl5*	GTAAAGCCCATGGAGTCCCA
A5_E4_F	mutant identification	*angptl5*	AATTCCTATCAAGCCAGGTGAA
A5_E4_R	mutant identification	*angptl5*	CCAAACGTTCCTCAAAATGTTT
A5_E3_gRNA	gene knockout	*angptl5*	AGCAAACCTCCAGCATACCA
A5_E3_F	mutant identification	*angptl5*	CACAAAGAACCAAGCAAAGAC
A5_E3_R	mutant identification	*angptl5*	AAGCCAGGTGAAGAAATACTG
dhrs9 qF	qRT-PCR	*dhrs9*	GCTGCATCTGGATGTGACTG
dhrs9 qR	qRT-PCR	*dhrs9*	ACTCGACCCTTGGCTTTCTT
tal1 qF	qRT-PCR	*tal1*	CTATTAACCGTGGTTTTGCTGG
tal1 qR	qRT-PCR	*tal1*	CCATCGTTGATTTCAACCTCAT
spi1b qF	qRT-PCR	*spi1b*	AGAGCTACAAAGCGTGCAGT
spi1b qR	qRT-PCR	*spi1b*	CCTGGGTCCATGAAATGG
gata1a qF	qRT-PCR	*gata1a*	GTCCAGTTCGCCAAGTTTAC
gata1a qR	qRT-PCR	*gata1a*	GGGTTGTAGGGAGAGTTTAG
18s rRNA qF	qRT-PCR	*rna18s*	TCGCTAGTTGGCATCGTTTATG
18s rRNA qR	qRT-PCR	*rna18s*	CGGAGGTTCGAAGACGATCA
etrsp qF	qRT-PCR	*etrsp*	GAGCTGTTGCACAAAGGTCA
etrsp qR	qRT-PCR	*etrsp*	CAGAGAGGGACGAGGTTCTG
F1	CHIP-qPCR	*dhrs9*	CAGAATTATTAGCCCCACTGAA
R1	CHIP-qPCR	*dhrs9*	CCCTAACCTGCCTAGTTAACCT
F2	CHIP-qPCR	*dhrs9*	CCCAATGTTTCTGATATGTGCGATA
R2	CHIP-qPCR	*dhrs9*	ACACTGAGAGGGAACACCAAAG
F3	CHIP-qPCR	*dhrs9*	TACCTCCTGTGACCTGTCTT
R3	CHIP-qPCR	*dhrs9*	CATCTCAAGTTTGGAGTTTGT
itga6l-gRNA	Gene Knockdown	*Itga6l*	GGAGCTGCGGTGCCCTCTAG
itgb5-gRNA rank1	Gene Knockdown	*Itgb5*	GGTCATGGAGAATGCCACTG
itgb5-gRNA rank2	Gene Knockdown	*Itgb5*	GGTATCAAGTCACTGAACGG
