# Supplementary material for: Angptl5 restricts primitive hematopoiesis by promoting retinoic acid signaling in zebrafish
Source: PLoS Biol. 2026 Jun 25;24(6):e3003858. doi: 10.1371/journal.pbio.3003858 (PMC13298773; doi:10.1371/journal.pbio.3003858)

Fig.4D

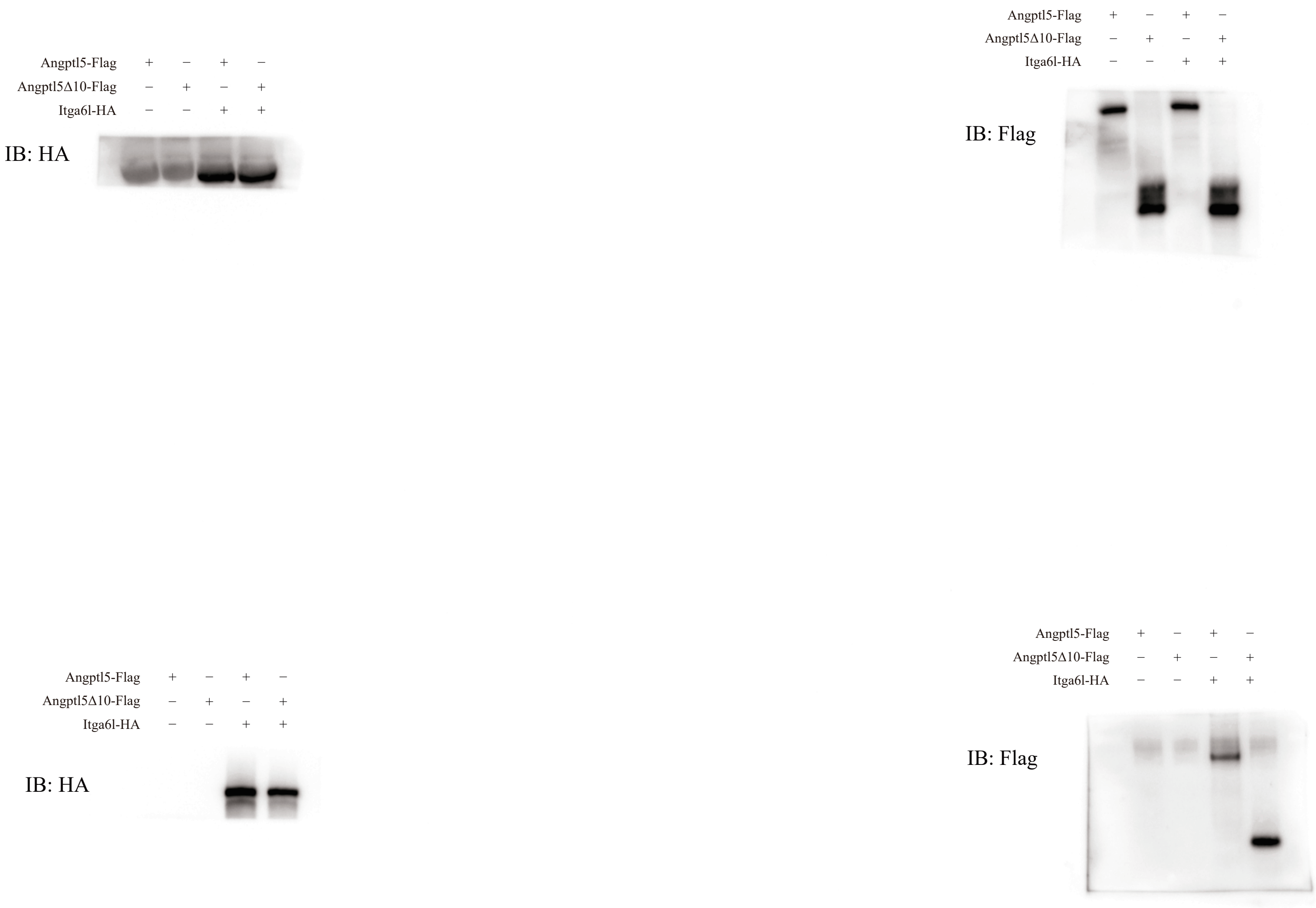
Angptl5-Flag + − + −
Angptl5Δ10-Flag − + − +
Itga6l-HA − − + +
IB: HA
Angptl5-Flag + − + −
Angptl5Δ10-Flag − + − +
Itga6l-HA − − + +
IB: Flag
Angptl5-Flag + − + −
Angptl5Δ10-Flag − + − +
Itga6l-HA − − + +
IB: HA
Angptl5-Flag + − + −
Angptl5Δ10-Flag − + − +
Itga6l-HA − − + +
IB: Flag

Fig.4F

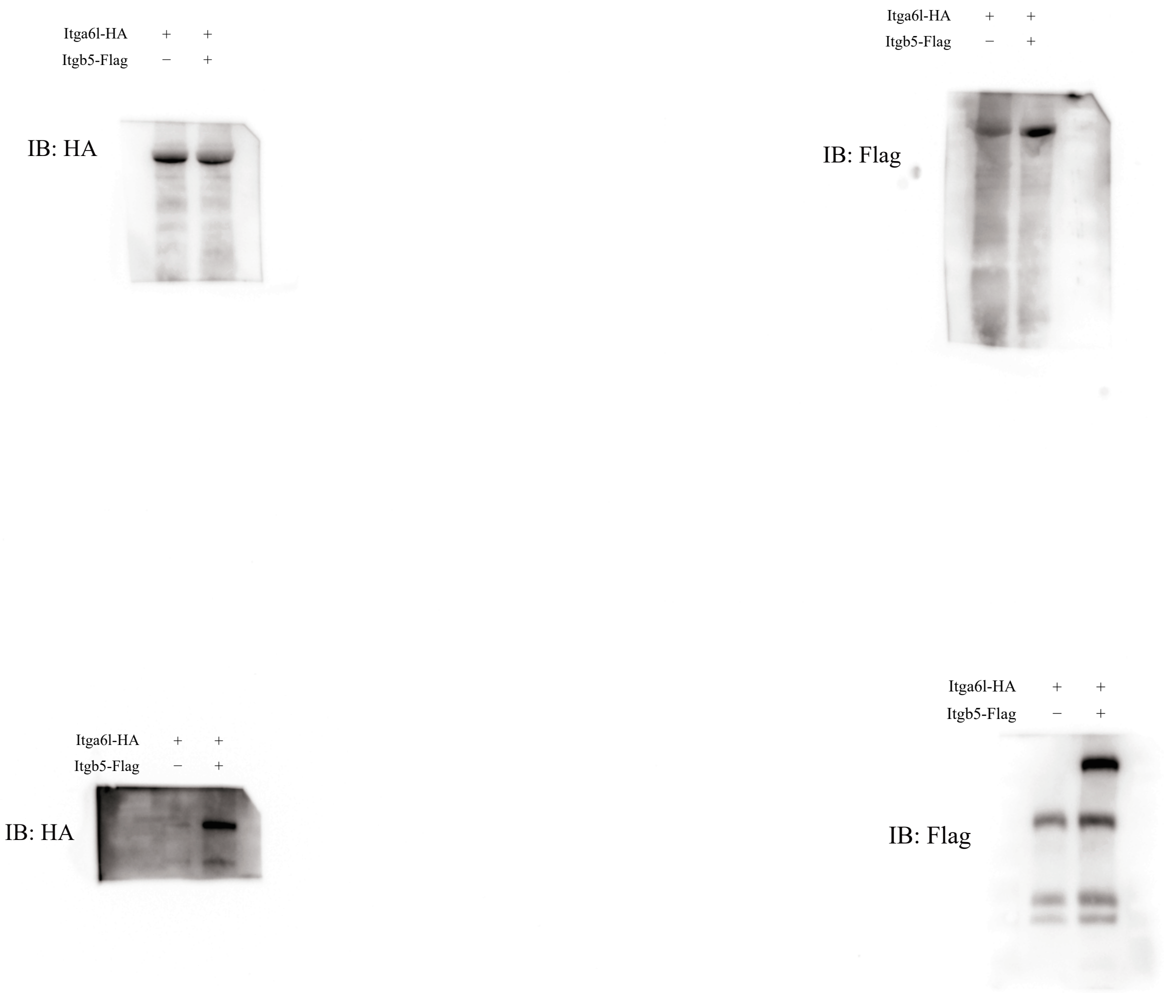

Itga6l-HA + +
Itgb5-Flag − +
IB: HA
Itga6l-HA + +
Itgb5-Flag − +
IB: Flag
Itga6l-HA + +
Itgb5-Flag − +
IB: HA
Itga6l-HA + +
Itgb5-Flag − +
IB: Flag

Fig.4I

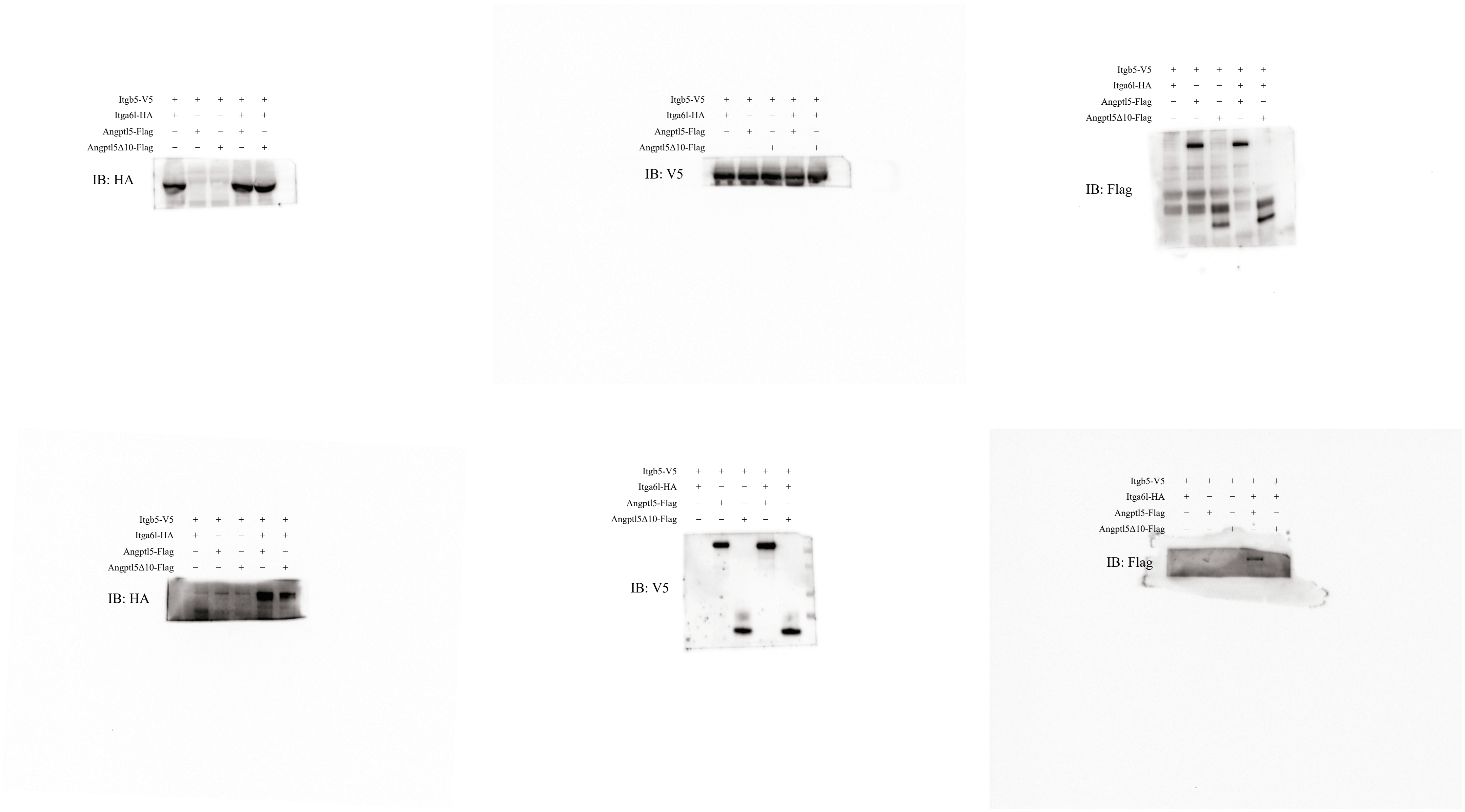
Itgb5-V5 + + + + +
Itga6l-HA + − − + +
Angptl5-Flag − + − + −
Angptl5Δ10-Flag − − + − +
IB: HA
Itgb5-V5 + + + + +
Itga6l-HA + − − + +
Angptl5-Flag − + − + −
Angptl5Δ10-Flag − − + − +
IB: V5
Itgb5-V5 + + + + +
Itga6l-HA + − − + +
Angptl5-Flag − + − + −
Angptl5Δ10-Flag − − + − +
IB: Flag
Itgb5-V5 + + + + +
Itga6l-HA + − − + +
Angptl5-Flag − + − + −
Angptl5Δ10-Flag − − + − +
IB: HA
Itgb5-V5 + + + + +
Itga6l-HA + − − + +
Angptl5-Flag − + − + −
Angptl5Δ10-Flag − − + − +
IB: V5
Itgb5-V5 + + + + +
Itga6l-HA + − − + +
Angptl5-Flag − + − + −
Angptl5Δ10-Flag − − + − +
IB: Flag

Fig.5E

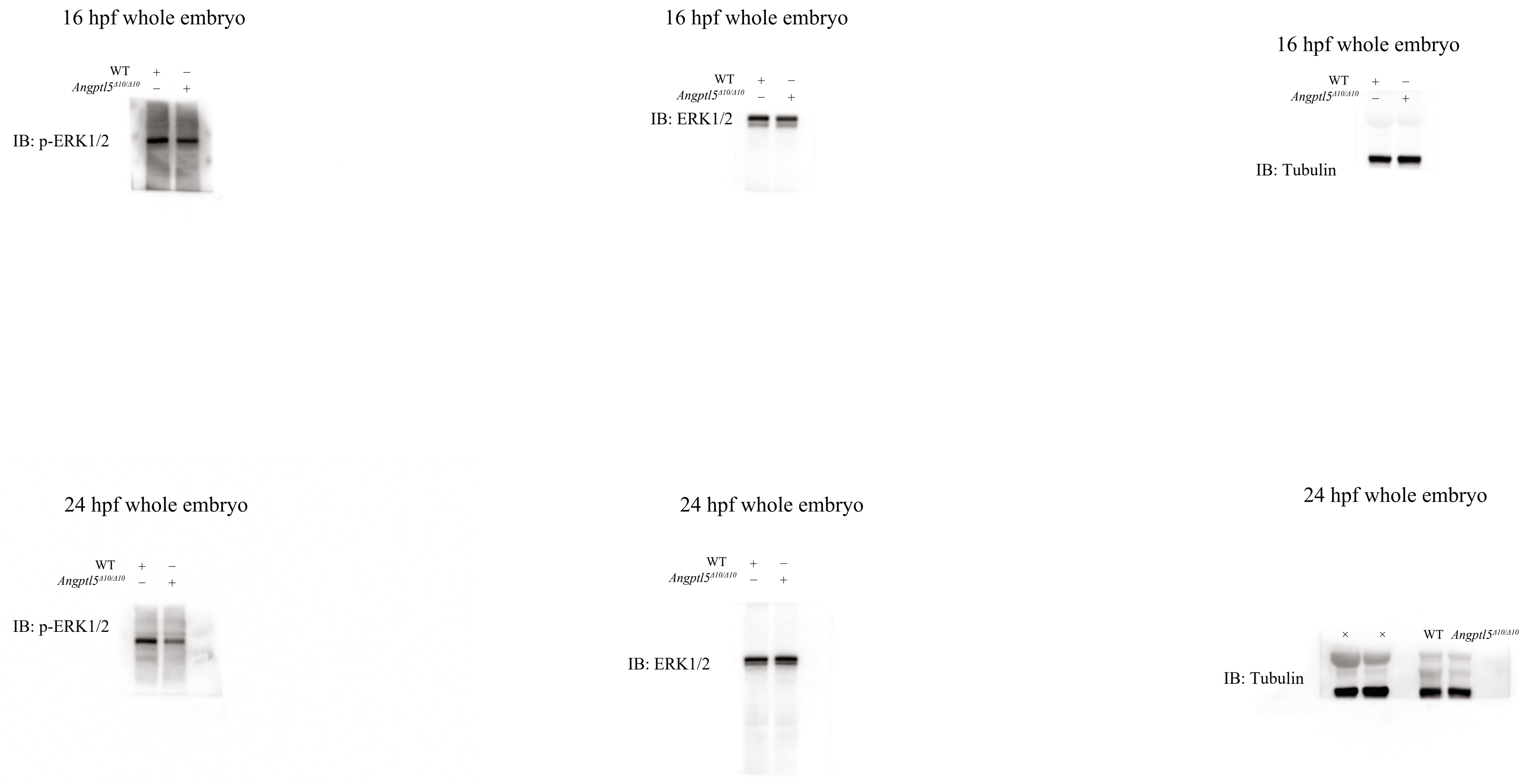
16 hpf whole embryo
WT + −
Angptl5$^{\Delta10/\Delta10}$ − +
IB: p-ERK1/2
16 hpf whole embryo
WT + −
Angptl5$^{\Delta10/\Delta10}$ − +
IB: ERK1/2
16 hpf whole embryo
WT + −
Angptl5$^{\Delta10/\Delta10}$ − +
IB: Tubulin
24 hpf whole embryo
WT + −
Angptl5$^{\Delta10/\Delta10}$ − +
IB: p-ERK1/2
24 hpf whole embryo
WT + −
Angptl5$^{\Delta10/\Delta10}$ − +
IB: ERK1/2
24 hpf whole embryo
× × WT *Angptl5*$^{\Delta10/\Delta10}$
IB: Tubulin

Fig.S8A

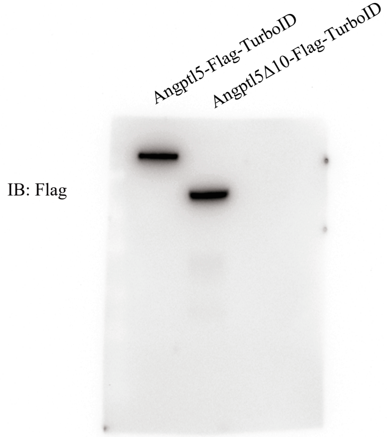

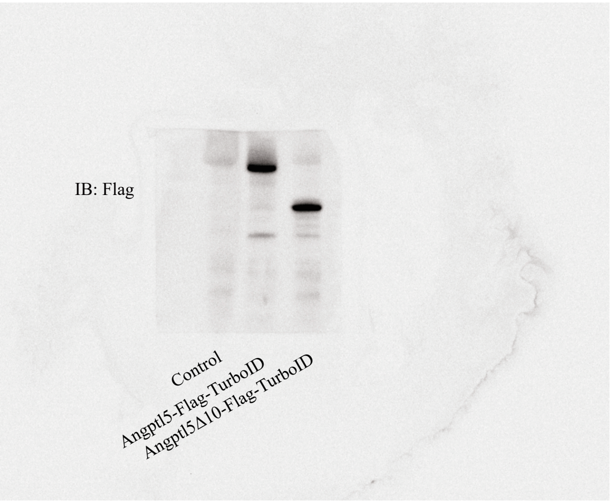

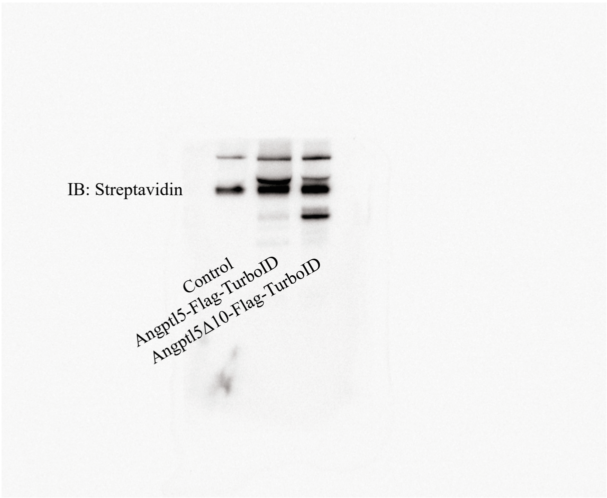

Fig.S11

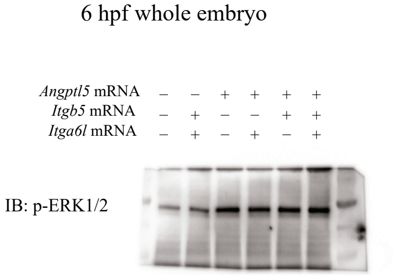

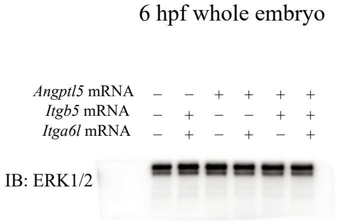

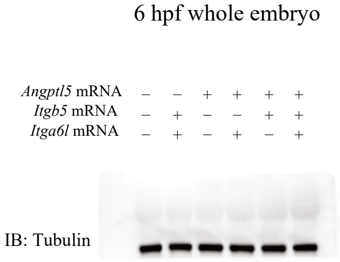

Supplement: S1 Raw Images — (PDF) [file pbio.3003858.s018.pdf]
